# Enhanced Chondrogenic Differentiation of Electrically Primed Human Mesenchymal Stem Cells for the Regeneration of Osteochondral Defects

**DOI:** 10.34133/bmr.0109

**Published:** 2024-12-18

**Authors:** Jongdarm Yi, Yujin Byun, Seong Soo Kang, Kyung Mi Shim, Kwangsik Jang, Jae Young Lee

**Affiliations:** ^1^ School of Materials Science and Engineering, Gwangju Institute of Science and Technology, Gwangju 61005, Republic of Korea.; ^2^ Department of Veterinary Surgery, College of Veterinary Medicine and BK21 FOUR Program, Chonnam National University, Gwangju 61186, Republic of Korea.; ^3^ Biomaterial R&BD Center, Chonnam National University, Gwangju 61186, Republic of Korea.

## Abstract

**Background:** Mesenchymal stem cells (MSCs) offer a promising avenue for cartilage regeneration; however, their therapeutic efficacy requires substantial improvement. Cell priming using electrical stimulation (ES) is a promising approach to augmenting the therapeutic potential of MSCs and has shown potential for various regenerative applications. This study aimed to promote the ES-mediated chondrogenic differentiation of human MSCs and facilitate the repair of injured articular cartilage. **Methods:** MSCs were subjected to ES under various conditions (e.g., voltage, frequency, and number of repetitions) to enhance their capability of chondrogenesis and cartilage regeneration. Chondrogenic differentiation of electrically primed MSCs (epMSCs) was assessed based on gene expression and sulfated glycosaminoglycan production, and epMSCs with hyaluronic acid were transplanted into a rat osteochondral defect model. Transcriptomic analysis was performed to determine changes in gene expression by ES. **Results:** epMSCs exhibited significantly increased chondrogenic gene expression and sulfated glycosaminoglycan production compared with those in unstimulated controls. Macroscopic and histological results showed that in vivo epMSC transplantation considerably enhanced cartilage regeneration. Furthermore, ES markedly altered the expression of numerous genes of MSCs, including those associated with the extracellular matrix, the Wnt signaling pathway, and cartilage development. **Conclusion:** ES can effectively prime MSCs to improve articular cartilage repair, offering a promising strategy for enhancing the efficacy of various MSC-based therapies.

## Introduction

The articular cartilage is a specialized connective tissue covering the bone ends in synovial joints; it provides a smooth, lubricated surface for low-friction articulation and serves as a shock absorber to distribute mechanical loads during movement [1]. Damage to the articular cartilage induces pain and swelling, which can lead to serious discomfort and eventual development of osteoarthritis (OA) [2]. Due to its complicated structure and the relatively low metabolic activity of chondrocytes, the articular cartilage exhibits a limited capacity for self-repair [3]. Autologous chondrocyte transplantation is currently the primary treatment for cartilage repair. For instance, the implantation of autologous chondrocytes with collagen membranes has demonstrated clinical improvement in the knee joints of patients [4]. However, autologous transplantation has notable limitations, such as the complex surgical procedure and challenges in mass production [5]. Hence, mesenchymal stem cells (MSCs) have garnered substantial attention in cell therapy as an alternative because of their ease of isolation from diverse sources (e.g., adipose tissue, umbilical cord blood, bone marrow, and Wharton’s jelly) and the lack of ethical issues. Additionally, their feasible in vitro proliferation, versatile differentiation potential, and abundant secretion of beneficial growth factors and cytokines make MSCs a promising source for articular cartilage regenerative therapy [6].

Many studies have used MSCs to treat articular cartilage defects. For instance, Park et al. [7] injected umbilical cord blood-derived MSCs with hyaluronic acid (HA)-based hydrogels into a knee full-thickness defect rat model and found enhanced cartilage repair in the hydrogel-only (MSC-free) and defect-only groups. However, the therapeutic efficacy of MSCs remains insufficient and requires additional strategies for improvement. Recently, cell priming or preconditioning has been suggested as a potent method to promote the therapeutic efficacy of MSCs for treatment of various injuries and diseases, including articular cartilage defects. For example, human synovium-derived MSCs cultured under hypoxic conditions showed enhanced colony-forming characteristics, up-regulated messenger RNA (mRNA) expression associated with chondrogenic differentiation, and increased glycosaminoglycan (GAG) levels in vitro [8]. Additionally, bone-marrow-derived MSCs exposed to dynamic compressive loading exhibited enhanced cartilage regeneration [9]. Various biochemical and biophysical stimuli, including cytokine treatment, 3-dimensional (3D) culture, and electrical stimulation (ES), have been employed to modulate the fate and enhance the therapeutic potential of MSCs [10].

Inherent bioelectrical signals are involved in various physiological functions, including intercellular transmission and reception of electrical signals [11]. Hence, ES has been increasingly explored to modulate the cellular metabolism, proliferation, migration, and differentiation of various cell types by influencing their intracellular pathways [12]. For example, 7- and 14-d ES significantly increased the osteogenic differentiation of rat bone-marrow-derived MSCs, enhancing the expression of type I collagen (COL1) alpha 1 chain, osteopontin, osterix, and calmodulin [13]. Several other studies also showed that ES-treated MSCs, even in the absence of exogenous growth factors, exhibited up-regulated gene expression and increased production of chondrogenesis-associated proteins at the cellular level [14]. Although the clear mechanisms underlying the effects of ES on MSC chondrogenesis have not been established, several mechanisms have been proposed. For example, ES could alter intracellular Ca^2+^ and adenosine triphosphate levels at the initial chondrogenic stages of MSCs and the enhanced secretion of various growth factors [15]. However, systematic effects of ES on the chondrogenic differentiation of MSCs in vitro and in vivo and comprehensive transcriptomic analyses remain unexplored.

**Fig. 1.**
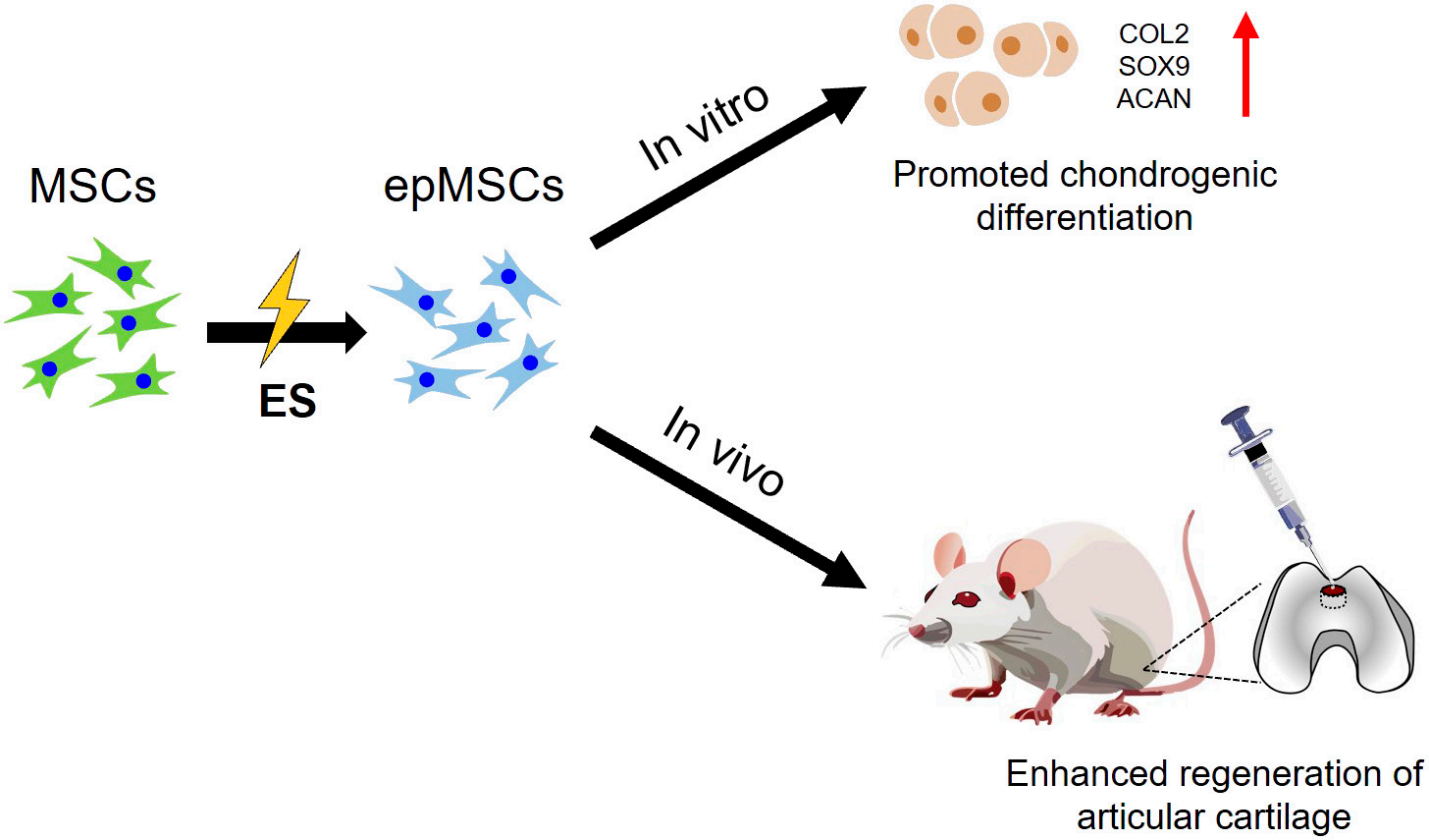
Schematic illustration of electrically primed mesenchymal stem cells (epMSCs) and their chondrogenic potential and regeneration ability in articular cartilage. MSCs, mesenchymal stem cells; ES, electrical stimulation; COL2, type II collagen; SOX9, SRY-box transcription factor 9; ACAN, aggrecan.

In this study, we aimed to enhance the chondrogenic differentiation of human MSCs by employing appropriate ES and facilitate the restoration of articular cartilage in vivo (Fig. 1). Specifically, we investigated the chondrogenic gene expression and sulfated GAG (sGAG) production in electrically primed MSCs (epMSCs). Additionally, epMSCs were transplanted with HA into the cartilage of rats with an osteochondral defect (OCD) to examine the regenerative capability of epMSCs. Finally, total RNA sequencing analysis was performed on MSCs and epMSCs to validate the ES-induced alterations in gene expression.

## Materials and Methods

### Materials

Human adipose-derived MSCs (hAD-MSCs) were purchased from PromoCell (Heidelberg, Germany). For ES, 316-L stainless-steel plates (3.8 cm × 2.8 cm × 0.7 cm) were obtained from BKSTEEL (Siheung, Republic of Korea). US origin sterile-filtered fetal bovine serum, papain from papaya latex (P3125), ascorbic acid, l-proline, dexamethasone, insulin–transferrin–selenium+3 (ITS+3) liquid medium supplement (I2771), glycine, sodium chloride (NaCl), chondroitin-4-sulfate A sodium salt from bovine trachea, and 1,9-dimethyl-methylene blue zinc chloride double salt (DMMB) were purchased from Sigma-Aldrich (St. Louis, Missouri, United States). Acetic acid (0.1 M) was purchased from Chem Lab (Zedelgem, Belgium). Human transforming growth factor beta 3 (TGF-β3) was purchased from GenScript (Piscataway, New Jersey, United States). Minimum essential medium-alpha, Dulbecco’s phosphate-buffered saline (DPBS), trypsin–ethylenediaminetetraacetic acid (EDTA) (0.05%), and antibiotic–antimycotic (100×) were purchased from Gibco (Grand Island, New York, United States). A WST-1 solution (EZ-Cytox) was purchased from DoGenBio (Seoul, Republic of Korea). TRIzol reagent and LIVE/DEAD viability/cytotoxicity kit for mammalian cells, mouse COL1 monoclonal antibody (MA1-26771), rabbit type II collagen (COL2) polyclonal antibody (PA1-26206), CD14–phycoerythrin (PE), CD34–PE, CD45–PE, CD73–PE, CD90–PE, CD105–PE, and mouse immunoglobulin G1 kappa isotype control–PE were purchased from Invitrogen (Carlsbad, California, United States). A hematoxylin and eosin (H&E) staining kit was purchased from BBC Biochemical (Mount Vernon, Washington, United States). Sodium hyaluronate (3 × 10^6^ Da) was purchased from LG Chem (Seoul, Republic of Korea).

### Cell culture and investigation of ES on MSCs

hAD-MSCs at passage 5 were used in all experiments. For maintenance, MSCs (1.0 × 10^4^ cells/cm^2^) were seeded on tissue culture plates and cultured in the growth medium (minimum essential medium-alpha supplemented with 10% fetal bovine serum and 1% antibiotic–antimycotic) in a humidified 5% CO_2_ incubator at 37 °C. The medium was replaced every 2 d. After reaching 80% to 90% confluency, MSCs were subcultured using 0.05% trypsin–EDTA at 37 °C for 5 min, centrifuged at 300×*g* for 5 min, and seeded onto the stainless-steel plates at a cell density of 1.5 × 10^4^ cells/cm^2^, followed by incubation at 37 °C for 24 h for subsequent culture.

ES of the MSCs was performed using a 2-electrode system consisting of a platinum mesh (5 mm × 10 mm) and a cell-seeded stainless-steel plate as the counter and working electrodes, respectively. A copper tape was attached to the outer wall of the plate to connect the electrodes with a multichannel potentiostat (VersaSTAT3; Princeton Applied Research, Oak Ridge, Tennessee, United States). Based on previous studies [16,17], ES condition (0.3 or 0.6 V of voltage, 1 or 100 Hz of frequency, 5-ms duration, and 2-h stimulation per day) was employed on days 1, 2, and 3. In total, 5 experimental groups were included as follows: (a) unstimulated, (b) 0.3 V and 1 Hz, (c) 0.3 V and 100 Hz, (d) 0.6 V and 1 Hz, and (e) 0.6 V and 100 Hz. Culture media were refreshed before the initial ES on day 1, and no further media were exchanged throughout the 3-d ES.

The cytocompatibility of MSCs subjected to different ES conditions was assessed using the LIVE/DEAD assay kit and metabolic activity measurements. Cell viability and metabolic activity were measured the following day of ES (on day 4 of the entire experimental period). The cells were stained using the LIVE/DEAD assay kit according to the manufacturer’s protocol. Briefly, a staining solution was prepared with 2 μM acetoxymethyl calcein and 4 μM ethidium homodimer-1 in DPBS. Next, 1 ml of the staining solution was added to each well and incubated at 37 °C for an additional 10 min. The cells were washed twice with DPBS, and fluorescence images were acquired using a fluorescence microscope (DMI3000 B, Leica, Germany). Viability was demonstrated as the percentage of live cells (green) relative to the total cell count (green and red). Metabolic activity was measured using EZ-Cytox according to the manufacturer’s protocol. Briefly, the WST-1 solution was mixed with fresh growth medium at a volume ratio of 1:10. The cells were then incubated in the mixed solution at 37 °C for an additional 1.5 h. The absorbance of the supernatant was measured at 450 nm using a microplate reader (Varioskan LUX; Thermo Fisher Scientific) and subtracted from the absorbance of the cell-free medium.

### Fluorescence-activated cell sorting analysis

One day after ES (on day 4 of the entire experimental period), MSCs were stained for stemness markers and analyzed by flow cytometry. Briefly, the cells were detached using 0.05% trypsin–EDTA, washed with DPBS, and stained with monoclonal antibodies against CD14–PE, CD34–PE, CD45–PE, CD73–PE, CD90–PE, and CD105–PE at 25 °C for 30 min. The isotype control cells were stained using mouse immunoglobulin G1 kappa isotype control–PE, washed twice with DPBS, and analyzed using a flow cytometer (FACSCanto II, BD Biosciences, San Jose, California). Population (%) and mean fluorescence intensity (MFI) were obtained using the FlowJo software (version 10.10.0). The MFI was normalized to that of the control group (MSCs).

### Chondrogenic differentiation of MSCs

The chondrogenic medium was prepared by adding 0.3 mM ascorbic acid, 0.35 mM l-proline, 0.1 μM dexamethasone, 1× ITS+3, and 10 ng/ml TGF-β3 in a high-glucose Dulbecco’s modified Eagle’s medium. One day after the final ES, MSCs were collected and subjected to chondrogenic differentiation. Briefly, 2 × 10^5^ cells from each group were transferred to a 15-ml conical tube and centrifuged at 300×*g* to obtain cell pellets. The culture medium was then replaced with the chondrogenic medium, and the pellets were further incubated in a humidified 5% CO_2_ incubator at 37 °C. The chondrogenic medium was replaced thrice a week during the whole 2-week differentiation period.

### Quantitative real-time polymerase chain reaction analysis

Quantitative real-time polymerase chain reaction (PCR) was performed after 14 d of culture in the chondrogenic medium. Total RNA was extracted from each group using the TRIzol reagent according to the manufacturer’s protocol. The quantity and purity of the extracted RNA were assessed using an ultraviolet–visible spectrophotometer (BioDrop Duo, BioDrop, United Kingdom). Complementary DNA (cDNA) was synthesized from the isolated mRNA using the High-Capacity cDNA Reverse Transcription Kit (Applied Biosystems) following the manufacturer’s protocol. Quantitative real-time PCR was performed using the Power SYBR Green PCR Master Mix and a StepOnePlus Real-Time PCR system (Applied Biosystems) according to the manufacturer’s protocol. The expression of human COL2 alpha 1 chain (COL2A1), SRY-box transcription factor 9 (SOX9), and aggrecan (ACAN) was normalized to that of glyceraldehyde-3-phosphate dehydrogenase (GAPDH). The primer sequences of the individual genes were as follows: GAPDH (forward [FW]: 5′-ATT TGG TCG TAT TGG GCG-3′; reverse [RV]: 5′-TGG AAG ATG GTG ATG GGA TT-3′), COL2A1 (FW: 5′-GCA CCT GCA GAG ACC TGA AAC-3′; RV: 5′-GCA AGT CTC GCC AGT CTC CA-3′), SOX9 (FW: 5′-AGG AAG CTC GCG GAC CAG TAC-3′; RV: 5′-GGT GGT CCT TCT TGT GCT GCA C-3′), and ACAN (FW: 5′-CTA CCG CTG CGA GGT GAT G-3′; RV: 5′-TCG AGG GTG TAG CGT GTA GAG A-3′).

### Quantification of the sGAG content

The sGAG content in the cells was quantified using the DMMB assay. After 2-week incubation in the chondrogenic differentiation medium, the cell pellets were collected and washed twice with DPBS. Thereafter, they were incubated in the 300 μl of papain digest solution (0.8% [w/v] sodium acetate, 0.4% [w/v] EDTA disodium salt, 0.08% [w/v] cysteine hydrochloric acid [HCl], and 0.5% [v/v] papain from papaya latex in 0.2 M sodium phosphate buffer) at 60 °C for 2 h. The DMMB solution was prepared by dissolving 3.04 g of glycine, 1.6 g of NaCl, 95 ml of 0.1 M acetic acid, and 16 mg of DMMB in 1 l of double-distilled water (DDIW). Standard solutions were prepared using chondroitin-4-sulfate at various concentrations (10, 5, 2.5, 1.25, and 0 μg/ml in DDIW). Next, 100 μl of the standard or each digested sample solution was transferred to a 96-well plate, and 100 μl of the DMMB solution was added to each well. Absorbance was immediately measured at 525 nm using a microplate reader.

The DNA content of the cells was quantified using the Quant-iT PicoGreen double-stranded DNA assay kit according to the manufacturer’s protocol. Briefly, a DNA standard solution (100 μg/ml) was diluted with 10 mM Tris-HCl–1 mM EDTA (TE) buffer to concentrations of 2, 0.2, 0.02, and 0 μg/ml. Subsequently, 100 μl of the digested solution of cell pellet or standard solution was transferred to a 96-well plate. The PicoGreen reagent was diluted with TE buffer at a ratio of 1:200 and added to each well. After 5-min incubation at 25 °C, the fluorescence intensity of the reaction mixture was measured using the microplate reader with emission and excitation wavelengths of 480 and 520 nm, respectively.

### Pellet histology analysis

The cell pellets were fixed with 3.7% formaldehyde for 30 min and washed twice with DPBS. Then, the samples were incubated in sucrose solution (30% [w/v] in DPBS) for 24 h and embedded in the optimal cutting temperature compound. The samples were stored at 4 °C for 24 h and frozen in a refrigerator at −80 °C. The frozen samples were sectioned into 5-μm thick slides using a cryotome (Leica, Germany). For Alcian blue staining, sample slides were washed with DDIW for 1 min, incubated in 3% acetic acid for 3 min, and incubated in the Alcian blue solution (pH 2.5) at 25 °C for 30 min. The tissue sections were gently washed under running tap water for 10 min, followed by 3 washes with DDIW. The sections were incubated in 95% ethanol for 1 min and dehydrated thrice with 100% ethanol. The samples were treated with xylene twice to remove the ethanol. Slides were mounted using Canada balsam and xylene mixtures prepared at a ratio of 1:1. For Safranin-O staining, the sections were washed in DDIW for 1 min and incubated in the Weigert’s iron hematoxylin solution for 5 min. Then, the slides were washed with DDIW 4 times and incubated in 1% (v/v) acetic acid for 30 s, followed by incubation in 1% (w/v) Safranin-O for 10 min. The slides were rinsed with 95% ethanol for 1 min and washed twice with 100% ethanol for 2 min each. The samples were treated with xylene twice to remove the ethanol. The slides were mounted using Canada balsam mixed with xylene at a ratio of 1:1. Images were acquired using a research slide scanner (VS200; OLYMPUS, Tokyo, Japan). The ImageJ software was used to calculate the average of intensity (AOI) of both Alcian blue and Safranin-O staining for quantitative analyses.

For immunocytochemistry, the sections were first washed in DDIW for 1 min and incubated in methanol for 20 min. The slides were then washed twice with DPBS for 5 min each. Following this, the slides were incubated in a permeabilization/blocking solution (0.1% [v/v] Triton X-100, 3% [w/v] bovine serum albumin in DPBS) at 25 °C for 1 h. After 2 washes with DPBS, the samples were incubated in the primary antibodies (anti-COL1 mouse monoclonal antibody and anti-COL2 rabbit polyclonal antibody, both diluted 1:100 in 3% [w/v] bovine serum albumin) at 25 °C for 1 h. The slides were subsequently washed twice with DPBS and incubated with secondary antibodies (Alexa Fluor 594 donkey anti-mouse immunoglobulin G (IgG) and Alexa Fluor 488 goat anti-rabbit IgG, both diluted 1:200 in DPBS) at 37 °C for 1 h. Finally, the slides were washed twice with DPBS for 5 min each, and fluorescence images were acquired using a VS200 research slide scanner (OLYMPUS, Tokyo, Japan) and analyzed using the ImageJ software.

### Establishment of the OCD model and surgical procedures

All animal experiments were approved by the Institutional Animal Care and Use Committee of Chonnam National University (CNU, Gwangju, Republic of Korea) (approval number: CNU IACUC-YB-2023-113), and the animals were cared for according to the Guidelines for Animal Experiments of the CNU. Sprague–Dawley rats (300 to 350 g, 10 weeks old) were divided into 4 groups (*n* = 5 per group). The details of the experimental groups are listed in the Table.

**Table.** Detailed experimental groups for animal studies using an osteochondral defect model

Group	Implanted material	Implanted cells
Control	No material	No cells
HA-only	2% HA	No cells
HA + MSCs	2% HA	2 × 10^5^ unstimulated MSCs
HA + epMSCs	2% HA	2 × 10^5^ epMSCs

HA, hyaluronic acid; MSC, mesenchymal stem cells; epMSCs, electrically primed mesenchymal stem cells

One day after the final ES, cells were detached using 0.05% trypsin–EDTA and centrifuged at 300×*g* for 5 min. Cells (1 × 10^7^ cells/ml) were then resuspended in 2% HA solution (w/v in DPBS).

The rat OCD model was established as previously described with slight modifications [7]. Animals were pre-anesthetized using 5 mg per kilogram of animal weight each of ketoprofen (EAGLE VET, Seoul, Republic of Korea), tramadol hydrochloride (JEIL PHARM., Seoul, Republic of Korea) via subcutaneous injection for pain management, and enrofloxacin (Bayer Korea Ltd., Seoul, Republic of Korea) via intramuscular injection for preventing infection and 1 mg of atropine (JEIL PHARM., Republic of Korea) per kg animal weight via subcutaneous injection for bradycardia prevention. Anesthesia was induced via intraperitoneal injection of 10 mg of xylazine (Bayer Korea Ltd., Seoul, Republic of Korea) and 80 mg of ketamine hydrochloride (Yuhan, Seoul, Republic of Korea) per kg animal weight.

The animal skin was disinfected with 10% povidone and alcohol, and a lateral parapatellar incision was made to expose the distal femoral trochlear groove surface. A full-thickness defect with a diameter of 2 mm and a depth of 2 mm was created at the trochlear groove using a surgical motor (Straumann Surgical Motor Pro Set 230 V; NSK, Tokyo, Japan) and a 2-mm trephine bur (JEUNG DO BIO & PLANT CO., Ltd., Seoul, Republic of Korea). The defect size was verified using a dental probe and surgical guide shaped with 3D-printed columns (diameter, 2 mm; depth, 2 mm). Subsequently, in each group except for the control, the sample (20 μl) was injected into the defect site using a 20-gauge catheter. Following suturing, the surgical site was disinfected with 10% povidone, and a dressing film (Tegaderm film, 6 cm × 7 cm, 3M, Seoul, Republic of Korea) was applied. Finally, all animals received oral medication daily for 1 week as follows: 5 mg of tramadol (Yuhan, Seoul, Republic of Korea) and 5 mg of enrofloxacin (Bayer Korea Ltd., Seoul, Republic of Korea) per kilogram of animal weight with 60 ml of water per rat. After 4 and 8 weeks, the animals were euthanized using CO_2_.

### Macroscopic evaluation of cartilage repair

Distal femurs, including the defect, were harvested after euthanasia at 4 and 8 weeks after surgery and transplantation for macroscopic evaluation and imaging. Cartilage regeneration was assessed macroscopically to determine the extent of articular cartilage repair. The assessment included the degree of defect repair, integration with the border zone, and overall macroscopic appearance using the International Cartilage Repair Society (ICRS) macroscopic evaluation system (Table S1). Five independent experimenters, who were blinded to the sample information, evaluated the samples to ensure the reliability and statistical significance of the assessments. According to the scoring system, with a maximum of 12 points, higher scores were assigned to characteristics indicative of greater regeneration of damaged cartilage.

### Micro-computed tomography analysis of subchondral bone repair

The harvested distal femurs were fixed in neutral-buffered 10% formalin. Micro-computed tomography (micro-CT) analysis was performed to verify the extent of new subchondral bone formation in vivo. The 3D and 2-dimensional (2D) reconstructions (transverse and middle sagittal planes) of the cross-sections of the centrally located cartilage defect were generated using a SkyScan 1273 ex vivo device (Bruker-CT, Kartuizersweg 3B, 2550 Kontich, Belgium). The x-ray source was configured to produce voxels measuring 15 μm at 50 kV and 80 μA. The exposure time was set to 3,450 ms with a frame average of 3. A 0.5-mm aluminum beam filtration filter was used. The data were acquired at intervals of 0.5° with rotation steps of up to 150°. Image slice reconstruction was performed using the NRecon version 2.2.0.6 software (Bruker, Kontich, Belgium) using the Feldkamp algorithm with beam correction applied. The settings were normalized across all images with individual fine-tuning to optimize the deconvolution quality. Fine-tuning parameters included smoothing set to 0, a ring artifact reduction of 4 to 6, and beam-hardening correction set at 20%. Reconstructed images were aligned using the SkyScan DataViewer software (Bruker-CT, Kartuizersweg 3B, 2550 Kontich, Belgium) to ensure consistent positioning of each bone with concurrent triaxial dataset saving. Manual segmentation and quantification of the total lesion/core volume and edema were performed using the CTAn version 1.20.8.0 software (Bruker, Kontich, Belgium). The subchondral bone and femoral trochlear groove were identified as the regions of interest for the articular defect area in the femoral trochlear groove. The images were saved as a separate dataset for further analysis using the SkyScan CTAn version 1.20.8.0 software. Additionally, high-resolution 3D reconstruction imaging of the entire bone, including the damaged parts, was performed. The reconstructed 3D images were aligned using the SkyScan CTvol software (version 2.3.2.1; Bruker, Kontich, Belgium) with the concurrent image data. Both 3D and 2D reconstructions of the cartilage defect center were generated by micro-CT. Qualitative analysis was performed using 3D reconstructions of the newly formed subchondral bone. Additionally, 2D cross-sectional images were used to qualitatively confirm the presence of newly formed subchondral bone at the defect sites.

### Histological analysis

Histological analysis of cartilage regeneration at the defect sites was performed using H&E, Masson’s trichrome, Alcian blue, and Safranin-O staining. Additionally, immunohistochemical (IHC) staining for COL2 was performed. The samples were decalcified in 20% EDTA disodium salt dihydrate solution (pH 7.4; Duksan, Ansan, Republic of Korea) at 25 °C for 21 d. Then, the samples were longitudinally embedded in paraffin wax (Surgipath Paraplast, Leica, Nussloch, Germany) and sectioned to 4 μm-thick slices. Deparaffinization, dehydration, and clearing of tissue slides were performed, followed by H&E staining using an automatic stainer (Leica Autostainer XL [ST5010], Leica, Nussloch, Germany). Triple staining using a Masson’s trichrome kit (trichrome stain kit, Masson, StatLab, Texas, United States) was performed to identify collagen deposition and interstitial fibrosis. An Alcian blue pH 2.5 kit (Alcian blue stain kit, Abcam, Cambridgeshire, United Kingdom) and 0.1% Safranin-O solution (Sigma-Aldrich) were used to analyze the GAG and proteoglycan contents in the cartilage, respectively. Additionally, 0.05% fast green (Sigma-Aldrich) and Weigert hematoxylin solutions (BioGnost, Zagreb, Croatia) were used to distinguish the surrounding tissues. The deparaffinized and dehydrated tissue slides were incubated in sodium citrate buffer (Fisher Scientific, Pittsburgh, United States) at 60 °C overnight for antigen retrieval. IHC staining was performed using VECTASTAIN Elite ABC-HRP Kit (rabbit IgG PK-6101, Vector Laboratories, Newark, United States). For qualitative assessment of COL2, COL2 staining was performed at a dilution ratio of 1:100 for 3 h. Counterstaining was performed using hematoxylin (hematoxylin G3; BioGnost, Zagreb, Croatia). Finally, images of the stained sections were acquired using a slide scanner (ZEISS Axio Scan.Z1, Carl Zeiss Microscopy GmbH, Jena, Germany). Safranin-O-stained images were used to analyze and quantify the cartilage regeneration capacity, and other staining images were used for qualitative analysis. The images of the region of interest were acquired at dimensions of 3,000 μm × 2,000 μm, centered on the defect, using the ZEN Microscopy software (ZEISS ZEN blue edition, ZEISS, Oberkochen, Germany).

To quantitatively evaluate the degree of histological cartilage repair, the sections were analyzed using the O’Driscoll histological cartilage repair scale (Table S2) based on Safranin-O-stained images. The hyaline cartilage, structural characteristics (such as surface irregularity, structural integrity, thickness, and bonding to the adjacent cartilage), freedom from cellular changes of degeneration, freedom from degenerative changes in adjacent cartilage, and reconstitution of subchondral bone were evaluated independently by 5 veterinarians blinded to the sample to ensure reliability and statistical significance of the measurements. The scoring system had a maximum score of 24 points, with higher scores indicating a greater degree of cartilage regeneration. The percentage of Alcian blue-, Safranin-O-, and COL2-positive areas was quantified by calculating the ratio of the positively stained area within the regenerated area to the total defect area in the OCD according to previous studies [18].

### Total RNA sequencing and data analysis

From each sample, 1 × 10^6^ MSCs and epMSCs were collected for total RNA sequencing. Quantitative RNA sequencing (Quant-Seq) was performed in duplicate (Ebiogen, Seoul, Republic of Korea). The results were analyzed using the ExDEGA software (Ebiogen, Seoul, Republic of Korea), as described previously [19]. Differentially expressed genes (DEGs) with fold changes ≥ 1.5, log_2_(normalized data) ≥ 4, and *P* values ≤ 0.05 were selected for further analyses. Relevant Gene Ontology (GO) analysis was performed using the DAVID (http://david.abcc.ncifcrf.gov/) and QuickGO databases (https://www.ebi.ac.uk/QuickGO/), with reference to 128 identified genes. Hierarchical clustering analysis was represented as a heat map using the MultiExperiment Viewer software (version 4.9.0; J. Craig Venter Institute, Rockville, Maryland, United States).

### Statistical analyses

All tests were performed in quadruplicate (*n* = 4) unless otherwise noted. Results are reported as mean ± standard deviation unless otherwise noted. Differences between groups were analyzed using one-way analysis of variance (ANOVA) followed by Tukey’s test used for comparisons, using the Origin software. Differences were considered significant at *P < 0.05 and **P < 0.01.

## Results

### Cytocompatibility of ES conditions

**Fig. 2.**
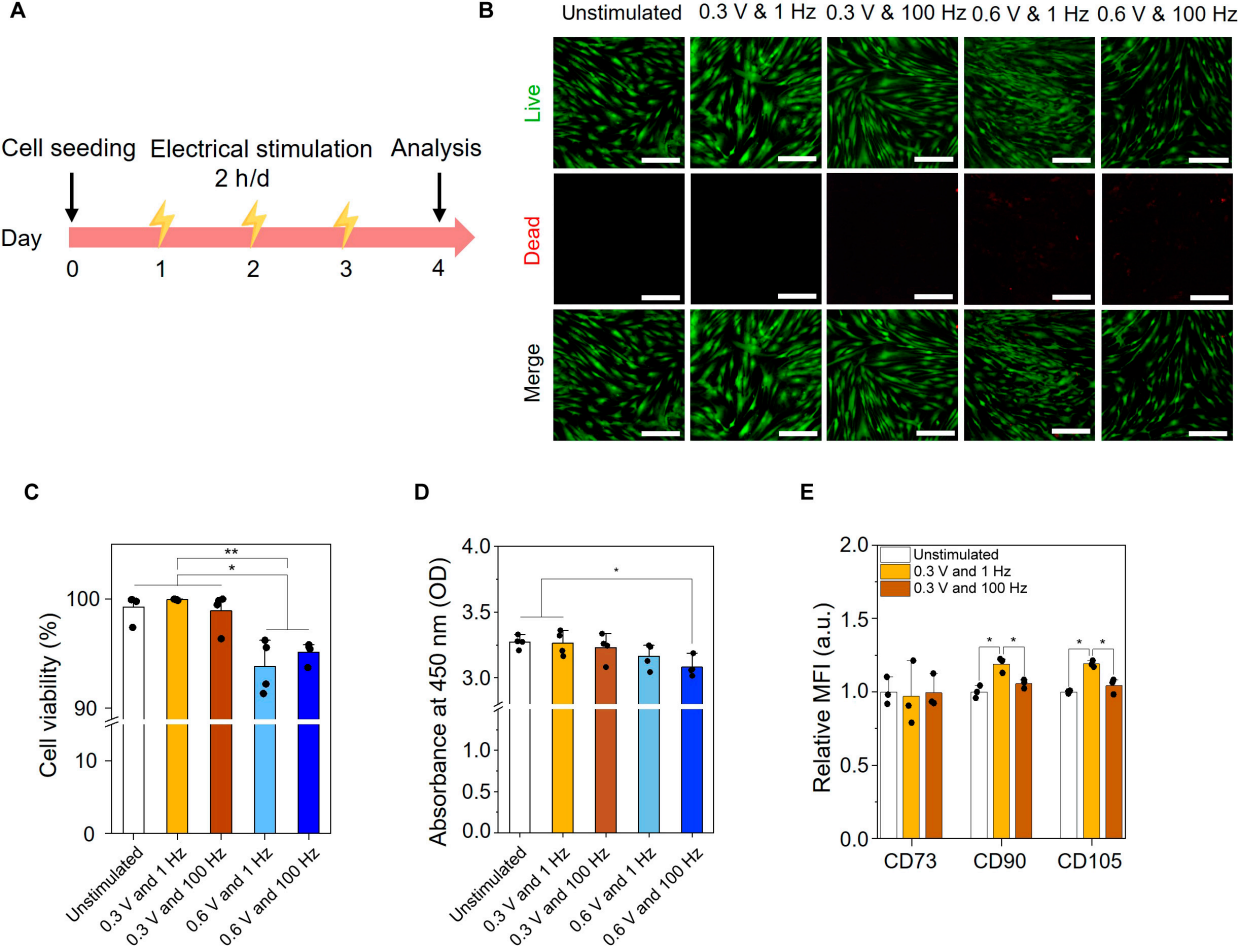
Viability and stemness maintenance of human adipose-derived MSCs electrically stimulated under diverse parameters. (A) Schematic experimental timeline. (B) Representative fluorescent images of MSCs and epMSCs. Scale bar = 100 μm. (C) Cell viability (*n* = 4). (D) Relative metabolic activities of MSCs and epMSCs (*n* = 4). (E) Mean fluorescence intensities (MFIs) of the positive marker (CD73, CD90, and CD105)-stained MSCs and epMSCs (*n* = 3). *P < 0.05; **P < 0.01. OD, optical density.

Appropriate ES is crucial for effective modulation of MSCs’ characteristics without damaging their viability [20]. To this end, we examined the ES parameters of various previously reported ES systems to induce the chondrogenic potential of MSCs while ensuring their viability and growth [21]. For example, the constant stimulation of human MSCs at 0.3 V and 1 Hz was reported to increase the expression of neurogenic markers (e.g., β3-tubulin and microtubule-associated protein 2) without adverse influence [22]. In this study, 2 different voltages (0.3 and 0.6 V) and 2 different frequencies (1 and 100 Hz) were employed, and the cell viability and metabolic activity were assessed on day 4 (Fig. 2A and Fig. S1). MSCs stimulated at 0.6 V exhibited significantly lower cell viability than the controls, regardless of the ES frequency (1 or 100 Hz) (Fig. 2B and C). For example, the viability of the MSCs stimulated at 0.6 V and 1 Hz and 0.6 V and 100 Hz were 93.8% ± 2.1% and 95.1% ± 0.8%, respectively. Additionally, MSCs stimulated at 0.6 V and 100 Hz (3.08 ± 0.06) exhibited significantly lower metabolic activity compared with that of both the unstimulated (3.27 ± 0.04) and 0.3 V and 1 Hz (3.26 ± 0.08) groups (Fig. 2D). No significant differences in cell viability or metabolic activity were observed between the unstimulated and ES groups (stimulated at 0.3 V [1 and 100 Hz]); hence, these 2 ES conditions were employed for subsequent experiments.

Moreover, ES-induced possible changes in stemness were investigated by analyzing the expression of stemness-related surface markers (Fig. 2E and Fig. S2). The stemness of MSCs is generally characterized by staining for positive (i.e., CD73, CD90, and CD105) and negative markers (i.e., CD14, CD34, and CD45) [23]. MSCs in the unstimulated and 2 ES groups exhibited a high population expressing positive markers (≥99%) and minimal population expressing negative markers (≤1%). This result suggests that ES did not alter the stemness characteristics of hAD-MSCs. Notably, MFIs of CD90 and CD105 were 1.2-fold higher in the epMSCs (0.3 V and 1 Hz) compared with those of unstimulated MSCs and epMSCs (0.3 V and 100 Hz). This increase in MFI implies that appropriate ES conditions can support the maintenance of stemness, which can further enhance the biological characteristics and/or therapeutic capabilities of MSCs, such as their immunomodulatory capacity and differentiation potential [24].

### In vitro chondrogenic differentiation of epMSCs

**Fig. 3.**
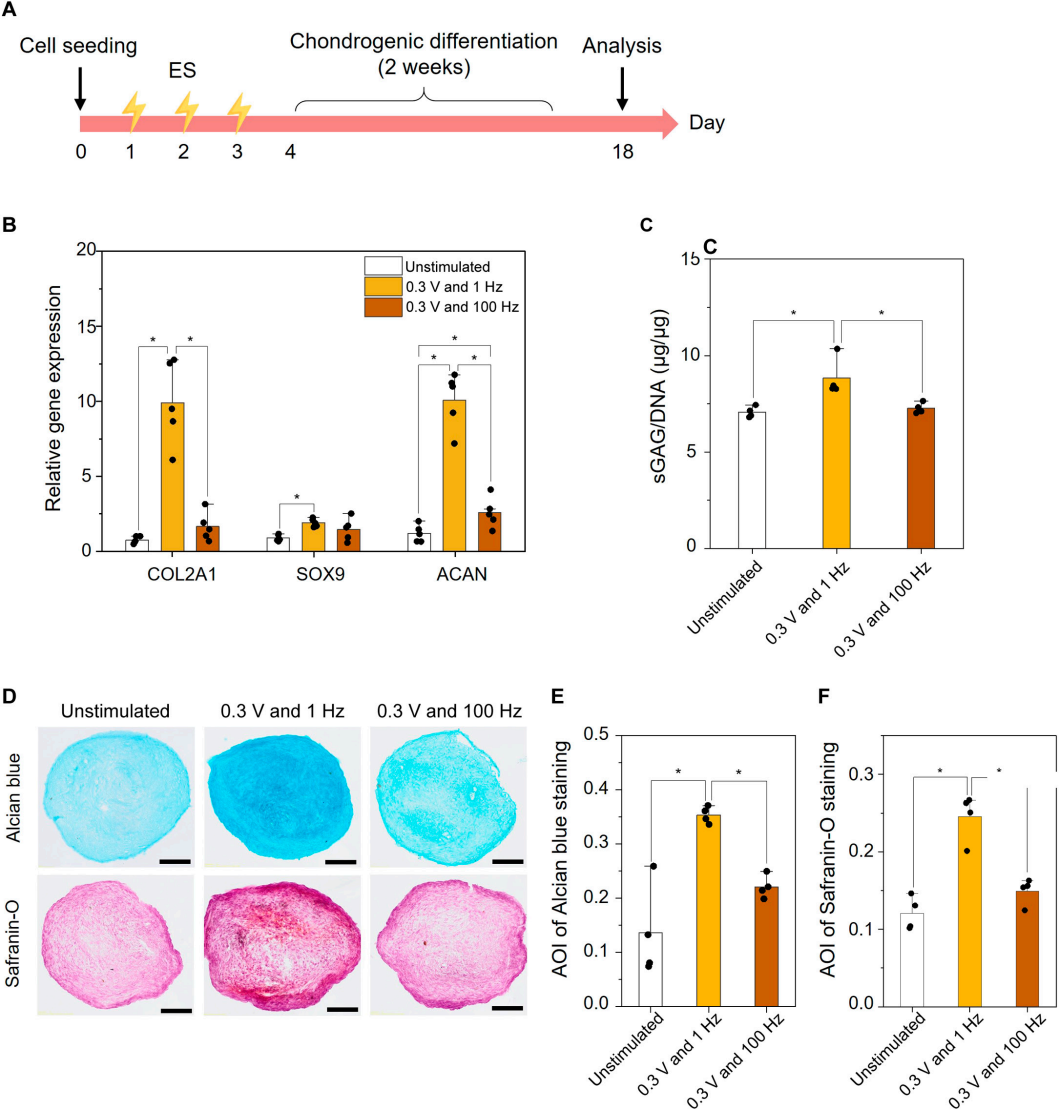
In vitro chondrogenic differentiation of epMSCs. (A) Schematic experimental timeline. (B) Relative expression of chondrogenic genes (COL2A1, SOX9, and ACAN) (*n* = 5). (C) Amount of sulfated glycosaminoglycan (sGAG) produced in each sample. The sGAG amount was normalized by DNA content in each sample. (D) Alcian blue and Safranin-O staining of MSC pellets. Scale bar = 100 μm. Average of intensity (AOI) values of (E) Alcian blue staining and (F) Safranin-O staining images in each group (*n* = 4). An asterisk (*) denotes a statistically significant difference (*P* < 0.05).

The chondrogenic differentiation capability of epMSCs was assessed by analyzing the mRNA expression and extracellular matrix (ECM) production after 2 weeks of culture in chondrogenic media (Fig. 3A). The expression of typical chondrogenic differentiation genes (i.e., COL2A1, SOX9, and ACAN) was the highest in the epMSC (0.3 V and 1 Hz) group compared with that in the unstimulated MSC and epMSC (0.3 V and 100 Hz) groups (Fig. 3B). COL2A1 is fibrillar collagen crucial for cartilage and involved in the early stages of chondrogenesis; it serves as one of the earliest markers indicating the commitment of cells to a chondrocyte lineage and the production of ECM necessary for cartilage structure [25]. In the epMSC (0.3 V and 1 Hz) group, COL2A1 expression was 13-fold and 6-fold higher than those in the unstimulated control and epMSC (0.3 V and 100 Hz) groups, respectively. SOX9 expression, a transcription factor for COL2 and crucial for chondrocyte differentiation and cartilage formation during the early and middle stages of chondrogenesis [26], significantly increased by 2.1-fold with ES at 0.3 V and 1 Hz compared with that in the unstimulated group. ACAN, a major proteoglycan involved in the middle to late stages of chondrogenesis, plays a critical role in mediating chondrocyte–ECM interactions [27]. ACAN expression in the epMSC (0.3 V and 1 Hz) group was 8.5- and 3.9-fold higher than those in the unstimulated control and epMSC (0.3 V and 100 Hz) groups, respectively. Interestingly, ES at 0.3 V and 100 Hz also led to a significant increase in ACAN expression but had no influence on COL2A1 and SOX9 expression.

The sGAG content, a major component of the cartilage ECM, was significantly increased by ES at 0.3 V and 1 Hz from 7.1 ± 0.3 to 8.8 ± 0.9, whereas the production of sGAG in epMSCs (0.3 V and 100 Hz; 7.3 ± 0.2) was similar to that in the unstimulated MSCs (7.1 ± 0.3) (Fig. 3C). Furthermore, GAG production in the pellets was histologically analyzed using Alcian blue and Safranin-O staining (Fig. 3D to F). Consistent with the sGAG quantification results, more intense staining of Alcian blue and Safranin-O was observed in the epMSC (0.3 V and 1 Hz) group than in the other groups. The AOI value was the highest in the epMSC (0.3 V and 1 Hz) group. The AOI values of unstimulated MSCs, epMSCs (0.3 V and 1 Hz), and epMSCs (0.3 V and 100 Hz) for Alcian blue staining were 0.14 ± 0.07, 0.35 ± 0.01, and 0.22 ± 0.02, respectively. For Safranin-O staining, the AOI values were 0.12 ± 0.02, 0.24 ± 0.03, and 0.15 ± 0.01 for unstimulated MSCs, epMSC (0.3 V and 1 Hz), and epMSC (0.3 V and 100 Hz), respectively. These results indicated substantial production of GAG in the epMSC (0.3 V and 1 Hz) group, which can be correlated with the significant increase in the expression of COL2A1, SOX9, and ACAN by ES in the 0.3 V and 1 Hz group. Therefore, MSCs electrically stimulated with 0.3 V and 1 Hz were denoted as epMSCs hereafter.

Immunofluorescence staining for COL1 and COL2 further verified the superior chondrogenic differentiation of the epMSCs compared to that of MSCs (Fig. S3). epMSCs exhibited higher expression of COL2 (a key marker of hyaline cartilage) and lower expression of COL1 (a key component of fibrocartilage) compared to MSCs. For example, COL2-positive areas, normalized by 4′,6-diamidino-2-phenylindole-positive cells, were 46.7% ± 8.2% and 23.2% ± 3.6% in the epMSC and MSC groups, respectively. Similarly, COL1-positive area was significantly lower in the epMSC group (24.8% ± 9.2%) than in the MSC group (48.9% ± 8.1%). These results further support the enhanced chondrogenic potential of epMSCs.

### Macroscopic evaluations of cartilage regeneration

**Fig. 4.**
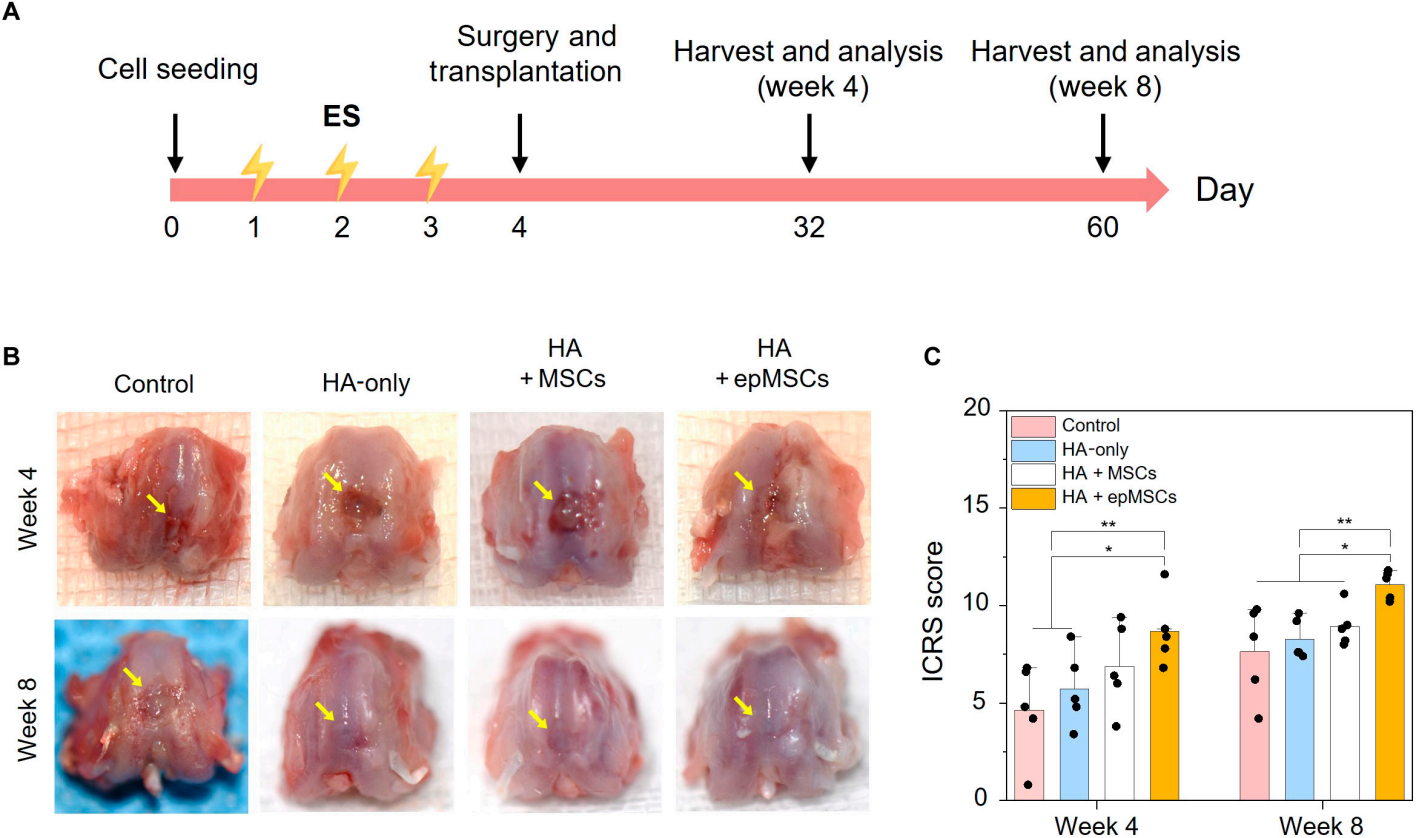
Macroscopic evaluation of cartilage repair in the rat model at 4 and 8 weeks after the surgery and treatments (nontreated control [Control], hyaluronic acid (HA)-only, HA + MSCs, and HA + epMSCs). (A) Schematic experimental timeline. (B) Gross images of osteochondral defects (OCDs) at 4 and 8 weeks after transplantation. The yellow arrow indicates the border between normal cartilage and regenerated cartilage. (C) Quantitative data of cartilage regeneration according to the scoring system of the International Cartilage Repair Society (ICRS) and macroscopic evaluation of cartilage repair at 4 and 8 weeks. An asterisk (*) denotes a statistically significant difference (*n* = 5, *P < 0.05; **P < 0.01).

The in vivo therapeutic efficacy of epMSCs for cartilage regeneration was investigated in a rodent OCD model (Fig. S4). The experimental groups included untreated controls, HA-only, HA + MSCs, and HA + epMSCs. At 4 and 8 weeks after the surgery and transplantation, distal femurs were harvested from each group, and the levels of regeneration were assessed (Fig. 4A). Inspection of the gross appearance of the femurs (Fig. 4B and Fig. S5) revealed that the HA + epMSCs group exhibited a superior recovery of the defect, better integration with the border zone, and smoother surfaces than those in the other groups. Additionally, the color of the regenerated cartilage in the HA + epMSCs group was similar to that of the normal cartilage, whereas it was distinctly different between the regenerated and normal cartilages in the control group. According to the ICRS evaluation (Fig. 4C), the average scores of the control, HA-only, HA + MSCs, and HA + epMSCs groups at week 4 were 4.6 ± 2.2, 5.7 ± 1.7, 6.9 ± 2.0, and 8.7 ± 1.6, respectively. Notably, the HA + epMSCs group exhibited significantly higher scores than the control and HA-only groups. At week 8, the ICRS scores of the control, HA-only, HA + MSCs, and HA + epMSCs groups were 7.6 ± 2.1, 8.3 ± 0.9, 8.9 ± 0.9, and 11.1 ± 0.6, respectively, in which HA + epMSCs still demonstrated the highest score. Altogether, the macroscopic evaluation indicated superior cartilage regeneration after epMSC transplantation.

### Micro-CT evaluation of the newly formed subchondral bone

**Fig. 5.**
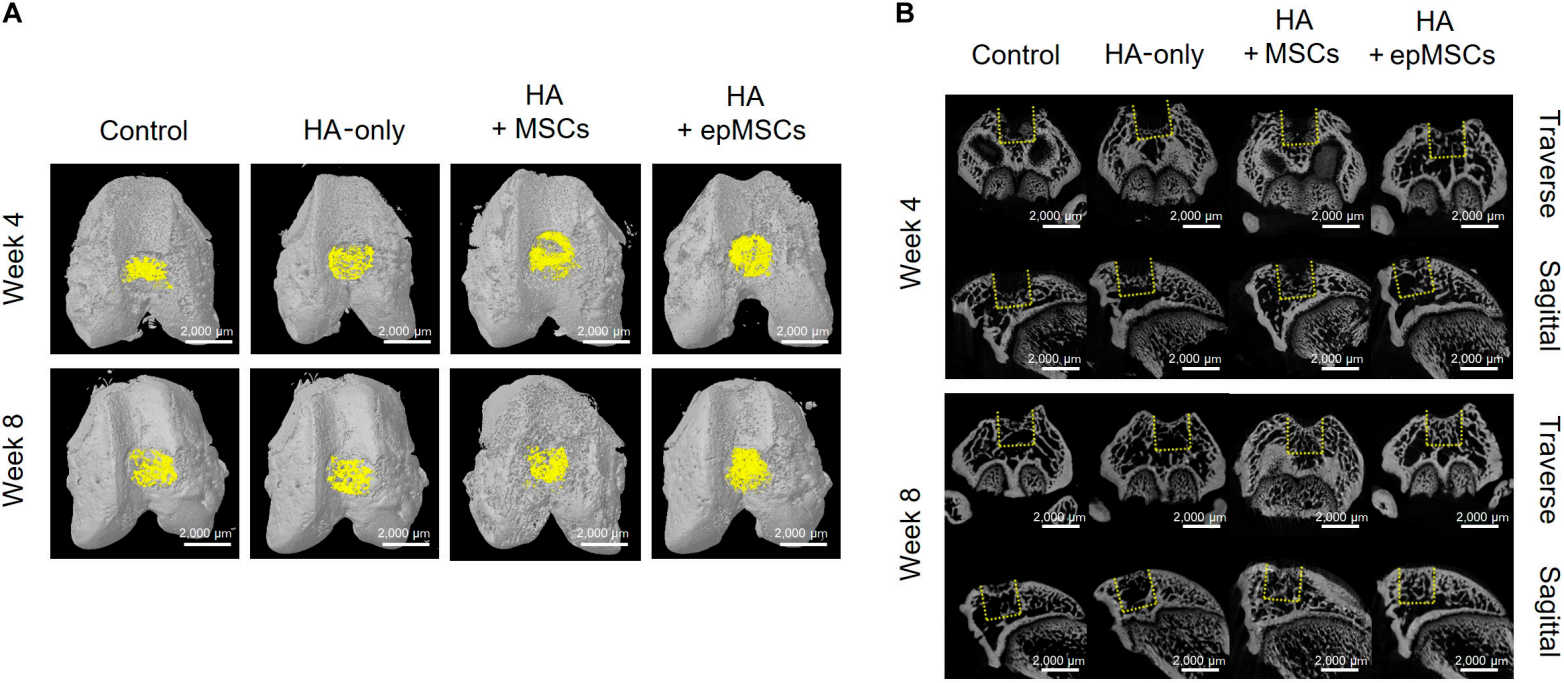
Micro-computed tomography (micro-CT) of the subchondral bone formation in the defect area (*n* = 5 for each group). (A) Three-dimensional (3D) reconstruction (yellow color indicates newly formed bone) and (B) 2-dimensional (2D) reconstruction in the cross-section of the articular joint at weeks 4 and 8 after transplantation. Indication of the original defect area with a dashed yellow box for distinguishing newly formed subchondral bone from existing bone. Scale bar = 2,000 μm.

Because the articular cartilage of a rat is typically thin and micro-CT imaging has limited resolution, it was challenging to accurately compare the degrees of cartilage regeneration among all groups using micro-CT images of rat cartilage. As an alternative, the samples were further qualitatively analyzed for newly formed subchondral bone using 3D and 2D micro-CT, as the OCD model in this study included parts of the subchondral bone and cartilage. In the 3D reconstruction images, the newly formed bone within the defect was highlighted in yellow (Fig. 5A). The 2D images provide cross-sectional views in the transverse and middle sagittal planes, centered on the defect (Fig. 5B). Overall, in both 3D and 2D images, the HA + epMSCs group presented less distinct borders between the defect and host bone and exhibited greater new subchondral bone formation within the defect than the other groups at both 4 and 8 weeks.

### Histological evaluations in the rat OCD model

**Fig. 6.**
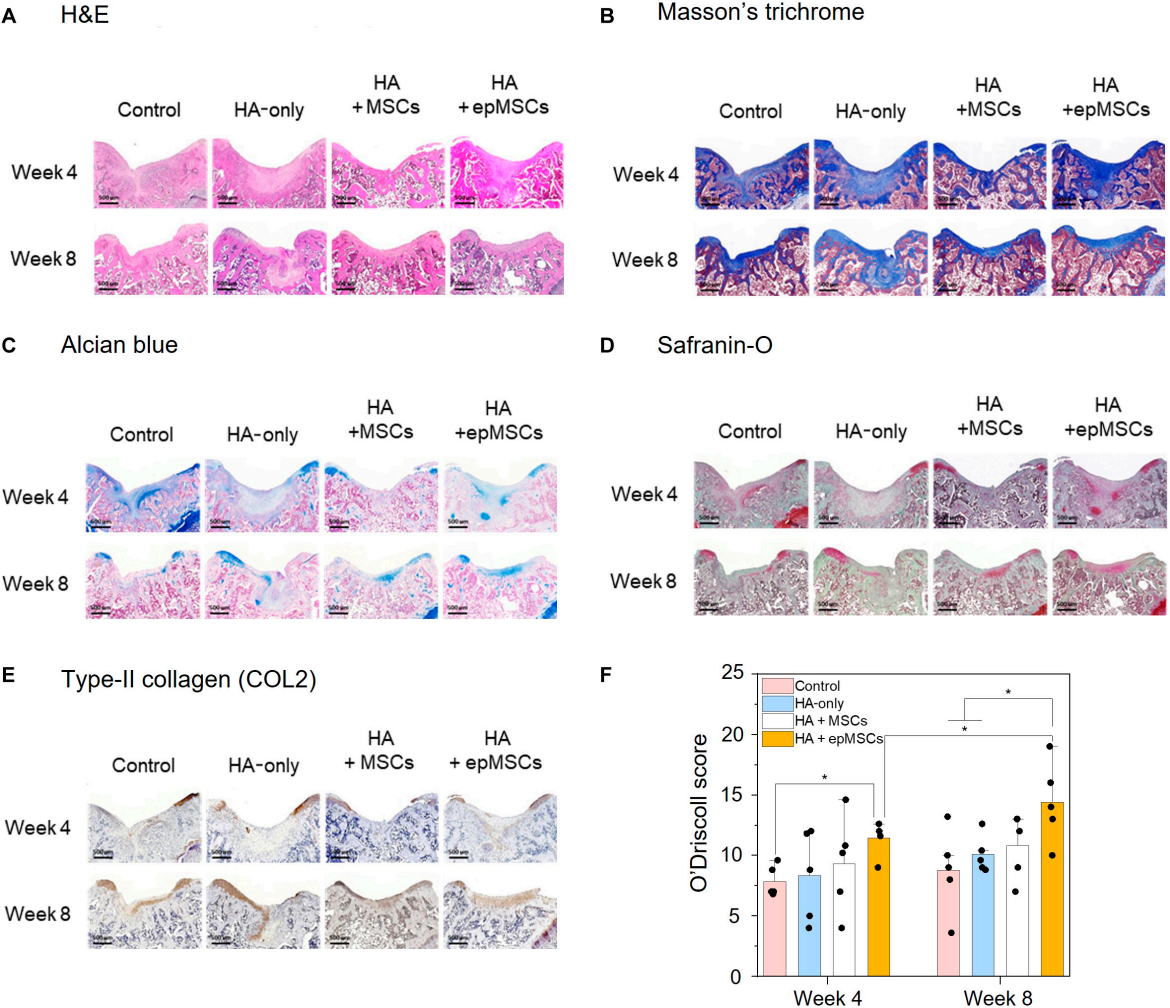
Histological and immunohistochemical (IHC) analyses of cartilage repair in the rat model at weeks 4 and 8 after transplantation. (A) Hematoxylin and eosin (H&E), (B) Masson’s trichrome, (C) Alcian blue, and (D) Safranin-O staining images and (E) type II collagen (COL2) IHC staining images of osteochondral defects at weeks 4 and 8 after transplantation. Scale bar = 500 μm. (F) Quantitative data of cartilage regeneration according to the O’Driscoll histological cartilage repair scale with Safranin-O stain at weeks 4 and 8. An asterisk (*) denotes a statistically significant difference (*n* = 5, *P < 0.05, **P < 0.01).

H&E staining (Fig. 6A) showed that the articular cartilage of the HA + epMSCs group presented a smooth and regular surface morphology compared with those of the other groups. Similarly, Masson’s trichrome staining revealed a darker blue hue in the HA + epMSCs group, suggesting a more abundant distribution of collagen fibers in the regenerated cartilage (Fig. 6B). Although the control group displayed faint Alcian blue and Safranin-O staining, the HA + epMSCs group showed distinct blue and orange–red staining (indicative of enhanced hyaline cartilage repair), increased proteoglycan levels, and calcified cartilage formation within the defect (Fig. 6C and D). Moreover, the regenerated cartilage in the HA + epMSCs group exhibited improved integration with the surrounding normal cartilage compared with that in the other groups. Additionally, IHC analysis revealed increased COL2 expression in the cartilage layer of the HA + epMSCs group compared with that in the other groups (Fig. 6E and Fig. S6). For quantitative analysis, Alcian blue, Safranin-O, and COL2 staining areas were calculated and compared (Fig. S7). At 4 weeks after transplantation, the staining areas were not significantly different among the groups; however, at 8 weeks after transplantation, the HA + epMSCs group demonstrated substantially larger staining areas for Alcian blue, Safranin-O, and COL2 compared to the other groups. These substantial increases in proteoglycan levels and collagen deposition suggest an enhanced ability of the epMSCs to promote cartilage regeneration.

Quantitative analysis using the modified O’Driscoll histological cartilage repair scale further indicated that the HA + epMSCs group exhibited a higher proportion of hyaline cartilage and a higher staining intensity of the matrix than the other groups (Fig. 6F). Although the integrity of the HA + epMSCs group was comparable to that of the other groups, this group presented a smoother surface morphology and thickness closer to that of normal adjacent cartilage. Notably, a relatively low tendency for hypocellularity and chondrocyte clustering was observed, indicating favorable regenerative processes within the group. The average O’Driscoll scores in the HA + epMSCs group at weeks 4 and 8 were 11.4 ± 1.3 and 14.4 ± 3.0, respectively, which were substantially superior to those of the other groups at both weeks 4 and 8. Interestingly, unlike the other groups, the HA + epMSCs group showed a significant regeneration at week 8 compared with that at week 4.

### Total RNA sequencing analysis of epMSCs

**Fig. 7.**
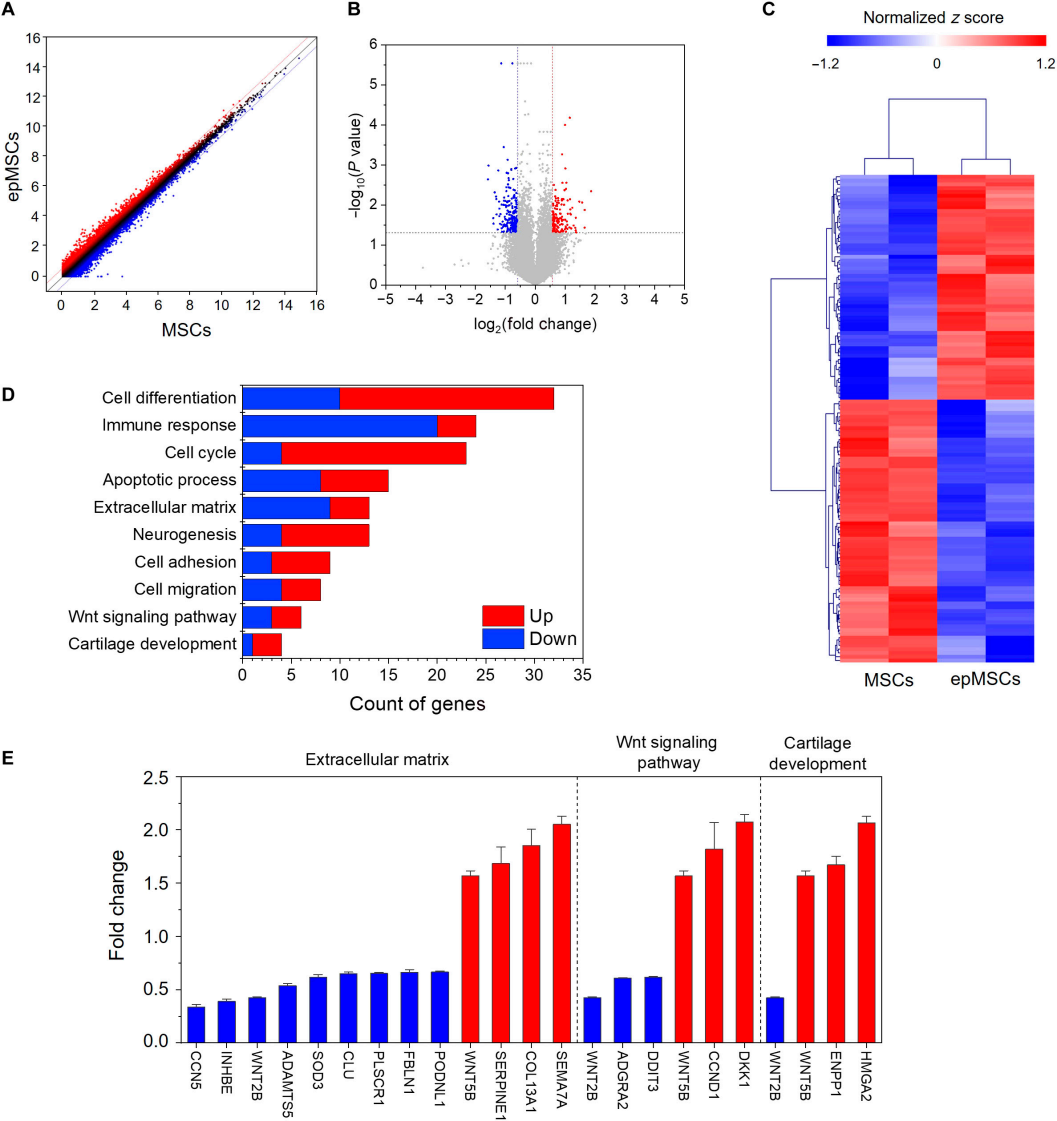
Total RNA sequencing of MSCs and epMSCs (*n* = 3 for each group). (A) Comparison of gene expression as a scatter plot between MSCs and epMSCs. (B) Volcano plot of −log_10_(*P* value) versus log_2_(fold change) in the expression of MSCs versus epMSCs. (C) Hierarchical clustering analysis based on the average gene expression compared by Euclidean distance coefficients. (D) Identification of the top 10 enriched Gene Ontology (GO) terms related to genes exhibiting significant expression changes induced by electrical stimulation. (E) Specific gene expression profiles of selected GO categories, including extracellular matrix, Wnt signaling pathway, and cartilage development. Red and blue colors indicate the up-regulation and down-regulation of each gene, respectively. HMGA2, high mobility group A2; ENPP1, ectonucleotide pyrophosphatase/phosphodiesterase 1; WNT2B, wingless-type mouse mammary tumor virus integration site; DKK1, Dickkopf-1; CCND1, cyclin D1; DDIT3, DNA damage-induced transcript 3; ADGRA2, adhesion G protein-coupled receptor A2; SEMA7A, semaphorin 7A; COL13A1, type XIII collagen alpha 1 chain; SERPINE1, serpin family E member 1; PODNL1, podocan-like protein 1; FBLN1, fibulin-1; PLSCR1, phospholipid scramblase 1; CLU, clusterin; SOD3, superoxide dismutase 3; ADAMTS5, a disintegrin and metalloproteinase with thrombospondin motifs 5; INHBE, inhibin subunit beta E; CCN5, cellular communication network factor 5.

Total RNA sequencing was performed to explore the mechanisms underlying epMSC-mediated enhancement of chondrogenic differentiation in vitro and cartilage regeneration in vivo. In total, 128 DEGs with a 1.5-fold or higher change (*P* < 0.05) compared with that in MSCs were identified in epMSCs (Fig. 7A and B). Out of the 128 DEGs induced by ES, 59 genes were up-regulated and 69 genes were down-regulated (Fig. 7C). GO categories were used to elucidate the DEG-associated biological processes. A total of 81 genes were classified within the top 10 identified biological processes, including cell differentiation, immune response, cell cycle, apoptotic process, ECM, neurogenesis, cell adhesion, cell migration, Wnt signaling pathway, and cartilage development (Fig. 7D). Regarding enhanced in vitro chondrogenic differentiation and in vivo cartilage regeneration, 3 biological processes (i.e., ECM, Wnt signaling pathway, and cartilage development) were further extracted (Fig. 7E).

The ECM plays a key role in forming a complex network of macromolecules, providing structural support, transducing signals for cellular communication, and regulating cellular behavior through interactions with soluble factors [28]. The articular cartilage function typically depends on its ability to maintain elasticity and high tensile strength, which are affected by the composition and quantity of ECM [29]. Therefore, alterations in ECM protein expression can considerably affect cartilage regeneration and function. In our results, out of the ECM-associated 13 genes, 9 genes (cellular communication network factor 5 [CCN5], inhibin subunit beta E [INHBE], wingless-type mouse mammary tumor virus integration site [WNT2B], a disintegrin and metalloproteinase with thrombospondin motifs 5 [ADAMTS5], superoxide dismutase 3 [SOD3], clusterin [CLU], phospholipid scramblase 1 [PLSCR1], fibulin-1 [FBLN1], and podocan-like protein 1 [PODNL1]) were down-regulated, whereas 4 genes (wingless-related integration site [WNT5B], serpin family E member 1 [SERPINE1], type XIII collagen alpha 1 chain [COL13A1], and semaphorin 7A [SEMA7A]) were up-regulated. In particular, FBLN1, COL13A1, PODNL1, INHBE, and SOD3 contribute to ECM organization, whereas ADAMTS5, CLU, SERPINE1, and WNT5B are involved in ECM remodeling [30–37]. CCN5, PLSCR1, SEMA7A, and WNT2B mediate cell–ECM interactions and signal transduction [36,38–40]. Notably, ES down-regulated ADAMTS5 (a key enzyme responsible for aggrecan cleavage) and up-regulated SERPINE1 (an inhibitor of proteases, such as tissue plasminogen activator) [35,41]. The Wnt signaling pathway is crucial for tissue development, including limb cartilage development, and regulating cellular behavior (e.g., proliferation and migration) [42,43]. Specifically, the canonical Wnt/β-catenin signaling pathway is known to regulate chondrocyte phenotype, maturation, and function during cartilage development [44]. Among the 6 Wnt-signaling-pathway-related genes, 3 genes were down-regulated (i.e., WNT2B, adhesion G protein-coupled receptor A2, and DNA damage-induced transcript 3 [DDIT3]) and 3 genes were up-regulated (WNT5B, cyclin D1, and Dickkopf-1 [DKK1]) in epMSCs. DDIT3 expression (a key mediator of Wnt signaling inhibition [45]) was down-regulated. Up-regulation was observed in WNT5B (highly expressed in chondrocytes and inhibiting chondrocyte hypertrophy [46]) and DKK1 (which inhibits OA cartilage destruction [47]). Regarding cartilage development, the expression of high mobility group A2 (HMGA2) (which stimulates chondrocyte proliferation [48]) and ectonucleotide pyrophosphatase/phosphodiesterase 1 (ENPP1) (involved in chondrocyte homeostasis [49]) was up-regulated in epMSCs. The analyses of DEGs and their roles in chondrocyte differentiation and cartilage development suggested that epMSCs may have a higher ability to differentiate into chondrocytes.

## Discussion

Owing to their unique ability to self-renew, differentiate, and modulate the immune response, MSCs have garnered considerable attention for the cell-based therapy of various tissue diseases, including OA [5]. However, their efficacy is frequently insufficient to achieve functional tissue regeneration, prompting efforts to potentiate MSCs by modulating their niches through external stimuli [11]. In this study, the effects of ES on the ability of MSCs to enhance chondrogenic differentiation and cartilage regeneration were investigated in an OA model, where natural regeneration is limited. While the inclusion of a positive control group, such as chondrocytes, could provide further insights, our primary aim was to investigate the enhanced effects of ES on MSC-based cartilage regeneration. As MSCs are more commonly used for regenerative medicine applications, the direct comparison between MSCs and epMSCs allows us to better assess the relative improvements in their therapeutic potential. Nevertheless, future studies may require comparison with other positive control groups, such as chondrocytes, to better demonstrate the therapeutic potential of epMSCs in cartilage repair.

Our findings indicated that priming MSCs with ES under optimal conditions (0.3 V and 1 Hz) significantly improved the expression of chondrogenic genes and cartilage-specific proteins, including COL2, and enhanced sGAG production compared with that in unstimulated MSCs, without damaging the stemness of MSCs. Furthermore, in the rat model of cartilage defects, epMSCs exhibited superior cartilage regeneration compared with that in the other groups, even in the absence of additional growth factor or predifferentiation treatment. Furthermore, RNA sequencing revealed significant changes in gene expression profiles after ES, particularly in pathways related to ECM organization, Wnt signaling, and cartilage development.

ES voltages exceeding 0.5 V can trigger undesirable electrochemical reactions within the culture media, leading to alterations in protein structures and disruptions to the cellular microenvironment [22]. Hence, we selected 0.3 V for ES of MSCs to avoid the possible adverse cellular damages and to encourage chondrogenic differentiation. In addition to voltage, we further explored the effects of different ES frequencies (1 and 100 Hz) on MSCs. ES at 1 Hz was found to significantly enhance chondrogenic differentiation of MSCs. Previous studies have also suggested that ES exerts frequency-dependent effects on cellular behaviors. For instance, ES at low frequency (2 Hz) led to significant increases in the vesicle mobility of astrocytes by >20%, whereas ES at higher frequencies (20 and 200 Hz) resulted in no significant change [50]. Another study also reported that ES frequencies between 1 and 2 Hz could increase the intracellular Ca^2+^ levels in C2C12 myotubes [51]. Due to the high electrical resistance of the cell membrane, low-frequency ES tends to remain at the cell surface rather than deeply penetrate the cell interior, potentially triggering biochemical signaling pathways through redistribution of cell surface receptors [52].

For OCD repair, numerous studies have used MSCs with hydrogels to enhance their applicability and efficacy [53]. In this study, we evaluated the effects of epMSCs using HA, a major ECM component of the cartilage. No statistically significant differences were observed between the HA + MSCs and HA-only groups. However, the HA + epMSCs group exhibited a significant increase in cartilage regeneration compared with that in the HA-only group. This difference suggests that preconditioning MSCs with ES significantly enhanced their regenerative potential. Several studies reported enhancement of chondrogenic differentiation of MSCs and cartilage regeneration by treatment with growth factors (e.g., TGF-β3) and prechondrogenic differentiation of MSCs before transplantation [20]. In contrast, the epMSCs developed in this study exhibited a high regeneration effect without complicated predifferentiation processes or additional growth factor treatment.

Nanosecond pulsed electric field can affect intracellular signaling pathways by activating c-Jun N-terminal kinase (JNK), p38, extracellular signal-regulated kinase, and Wnt signaling [54]. Recently, the JNK, cyclic adenosine monophosphate response element binding protein (CREB), and signal transducer and activator of transcription 3 (STAT3) signaling pathways were found to be associated with MSC chondrogenesis and OCD repair by stimulation with nanosecond pulsed electric field [20]. Our RNA sequencing analysis revealed GO changes in epMSCs, which can be further correlated with the observed improvement in chondrogenesis and cartilage regeneration. The increased expression of ECM-associated genes is consistent with the observation of increased sGAG and ECM production in epMSCs in the biochemical assays of this study (Fig. 3). Alterations in the gene expression in ECM, Wnt signaling pathway, and cartilage development revealed their roles in mediating the beneficial effects of ES on MSCs for cartilage regeneration without additional differentiation factor treatment. Additionally, ES was found to affect the cell cycle and apoptotic processes, subsequently affecting cellular proliferation and survival, which are essential for tissue growth and repair. DEGs involved in the immune response, cell differentiation, and other related processes suggest that ES can induce the creation of a favorable microenvironment for tissue regeneration. Despite the promising results of epMSCs for cartilage regeneration, further studies are necessary for their clinical translation. In particular, long-term studies are required to ensure the safety, efficacy, and functional stability of the regenerated cartilage. Additionally, further optimization of ES conditions for epMSCs is needed. The large-scale production of epMSCs must also ensure consistent quality and therapeutic efficacy, which is essential to meet regulatory refinements. Finally, the cost and scalability of epMSC-based therapies need to be addressed to make them accessible to a broader range of patients.

With an aim to improve therapeutic effectiveness of MSCs, we explored preconditioning MSCs with the appropriate ES in vitro to increase their chondrogenic differentiation. Transplantation of epMSCs with HA leads to superior cartilage regeneration and integration compared with HA-alone or HA–MSC treatments. According to the transcriptomic analysis, ES significantly alters the expression of many genes, including those associated with cell differentiation, ECM, Wnt signaling pathway, and cartilage development. Altogether, this study successfully demonstrated that epMSCs can serve as an effective platform for enhancing the efficacy of MSC-based therapy for various diseases, including cartilage regeneration.

## Data Availability

The data that support the findings of this study are available from the corresponding authors upon reasonable request.
