# Supplementary material for: Enhanced Chondrogenic Differentiation of Electrically Primed Human Mesenchymal Stem Cells for the Regeneration of Osteochondral Defects
Source: Biomater Res. 2024 Dec 18;28:0109. doi: 10.34133/bmr.0109 (PMC11654951; doi:10.34133/bmr.0109)
Supplement: Supplementary 1 — Figs. S1 to S7 Tables S1 and S2 [file bmr.0109.f1.docx]

Supplementary Information

Enhanced chondrogenic differentiation of electrically-primed human mesenchymal stem cells and therapeutic effects in osteochondral defect

*Jongdarm Yi^1^, Yujin Byun^2,3^, Seong Soo Kang^2,3^, Kyung Mi Shim^2,3^, Kwangsik Jang^2,3*^, and Jae Young Lee^1*^*

^1^School of Materials Science and Engineering, Gwangju Institute of Science and Technology, Gwangju, 61005, Republic of Korea

^2^Department of Veterinary Surgery, College of Veterinary Medicine and BK21 FOUR Program, Chonnam National University, Gwangju 61186, Republic of Korea

^3^Biomaterial R&BD Center, Chonnam National University, Gwangju 61186, Republic of Korea

**
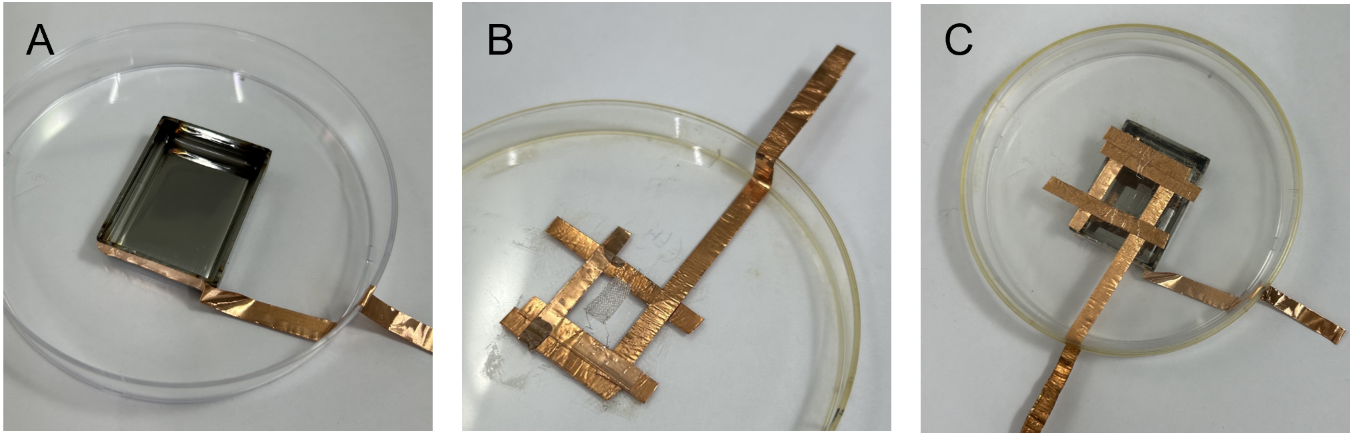
**

**Fig. S1.** Photographs of the lab-established electrical stimulation (ES) system. (A) SUS316L stainless steel plate used as a working electrode. (B) Platinum mesh used as a counter electrode. (C) Combined setup showing the stainless steel plate and platinum mesh in their positions for ES.

**
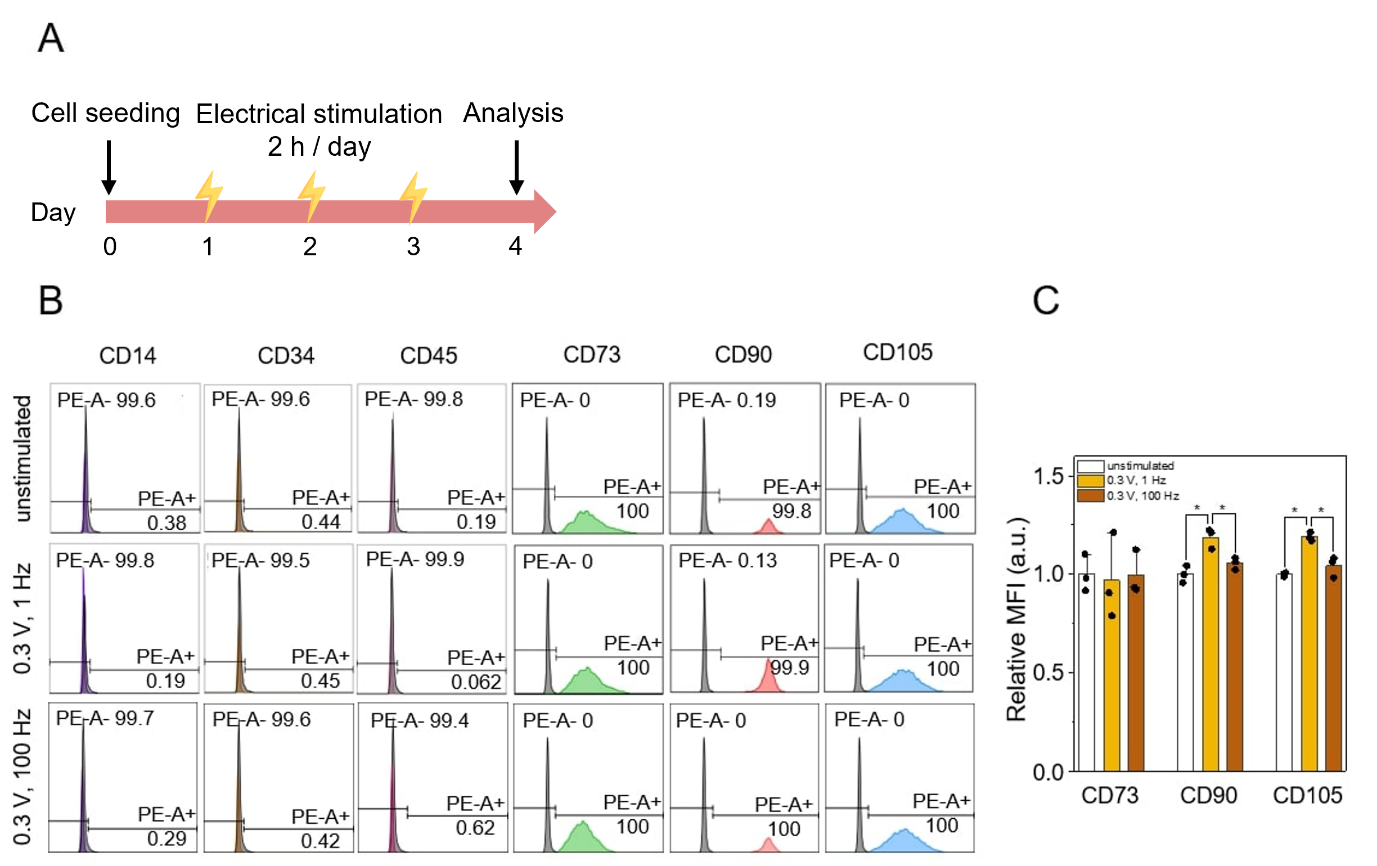
**

**Fig. S2.** Effects of electrical stimulation (ES) on stemness of human adipose-derived mesenchymal stem cells (MSCs). (A) Schematic representation of experimental timeline. (B) Flow cytometry of the MSCs and electrically primed MSCs (epMSCs) stained for negative markers (CD14, CD34, and CD45) and positive markers (CD73, CD90, and CD105).

**
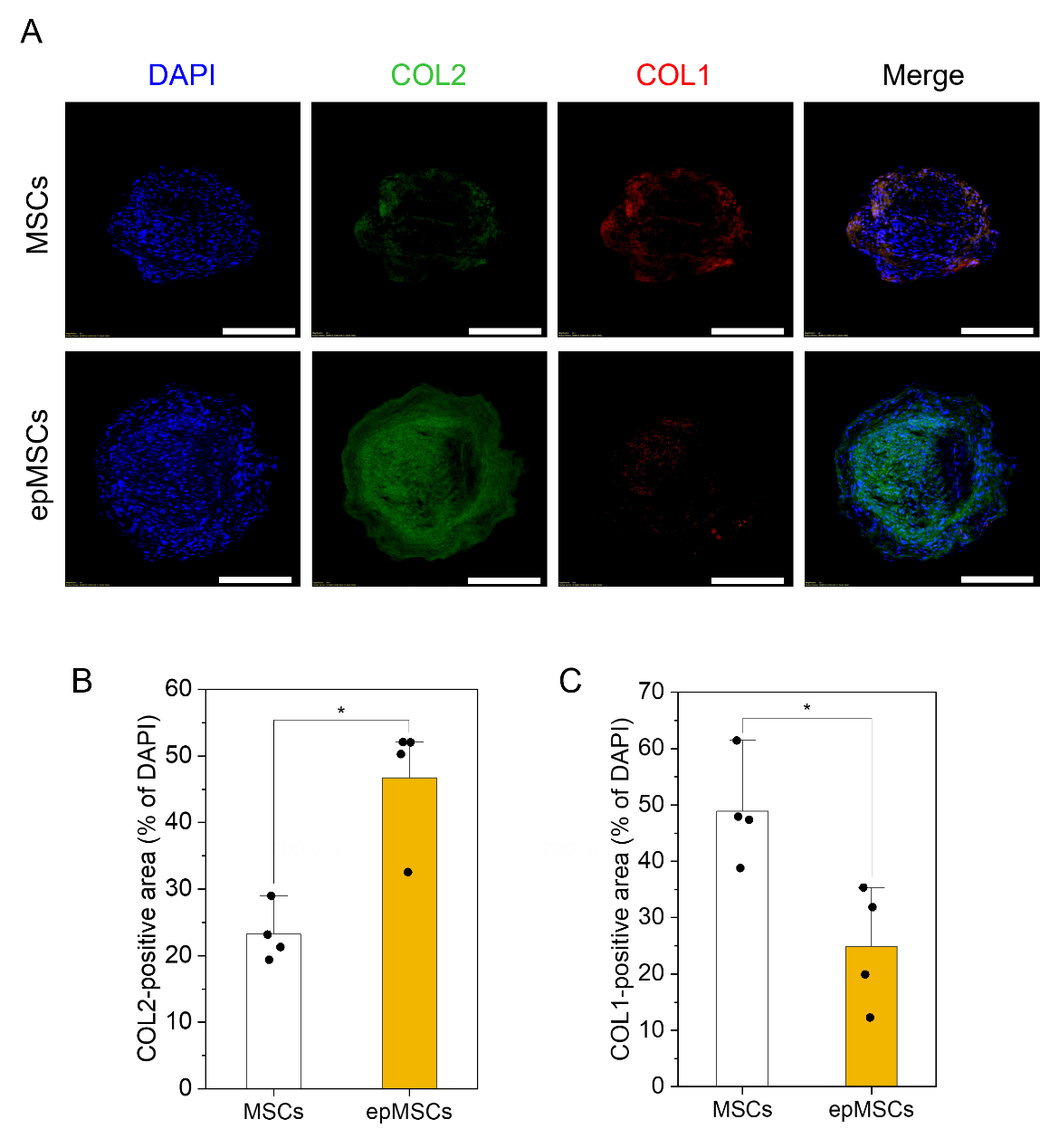
**

**Fig. S3.** Histological analysis of chondrogenic differentiation in vitro. 4',6-diamidino-2-phenylindole (DAPI) is used to label the nuclei. (A) Immunofluorescence staining of COL1 and COL2 in MSC and epMSC pellets after 2 weeks of chondrogenic differentiation. (B) Quantification of the COL2-positive area, normalized by the DAPI-positive areas in each group (*n* = 4, **p* < 0.05). (C) Quantification of the COL1-positive area, normalized by the DAPI positive areas in each group (*n* = 4, **p* < 0.05). Scale bar = 200 μm.

**
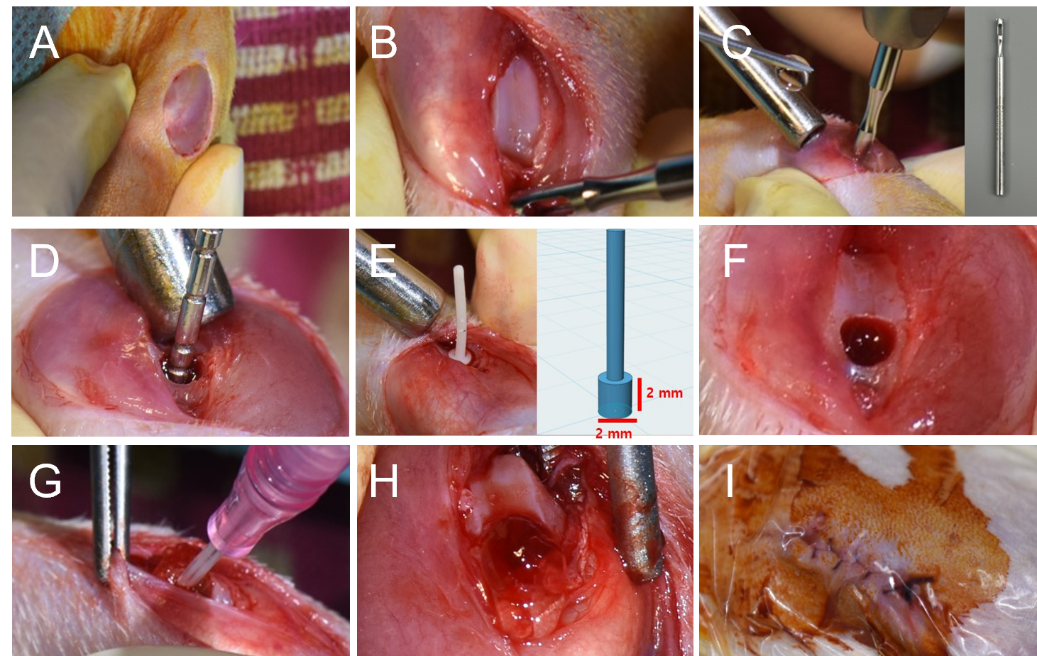
**

**Fig. S4.** Surgical procedures for the rat cartilage osteochondral defect model and transplantation of MSCs. (A) The lateral parapatellar incision. (B) Exposure of the distal femur trochlear groove. (C) Creation of the full-thickness defect (diameter 2 mm × depth 2 mm) at the trochlear groove using a surgical motor and trephine bur (diameter 2 mm). (D) Verification of the created defect size using the dental probe. (E) Verification of the created defect size using the three-dimensional printed column-shaped (diameter 2 mm × depth 2 mm) surgical guide. (F) Created defect. (G) Injection of the 20 μL of the sample into the defect using a 20-gauge catheter, except for the control group. (H) Confirmed sample injection into the defect. (I) The surgical site disinfected with 10% povidone and Tegaderm film application after the suture.


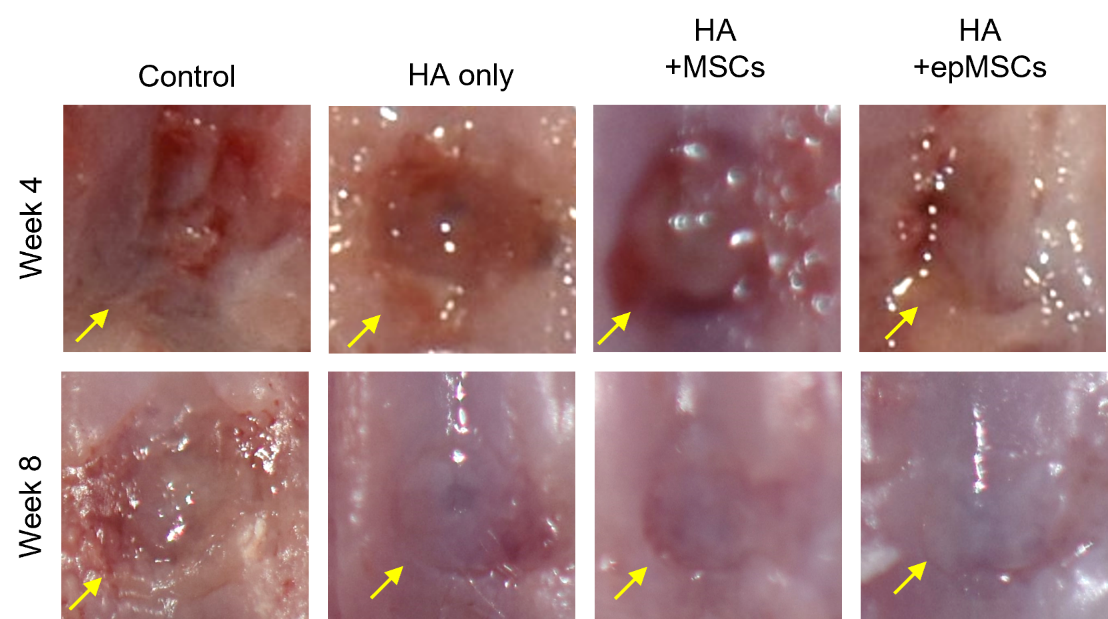


**Fig. S5.** Higher-magnification images of the defect area of macroscopic images at 4 and 8 weeks after transplantation, with yellow arrows indicating one side of the defect boundary.


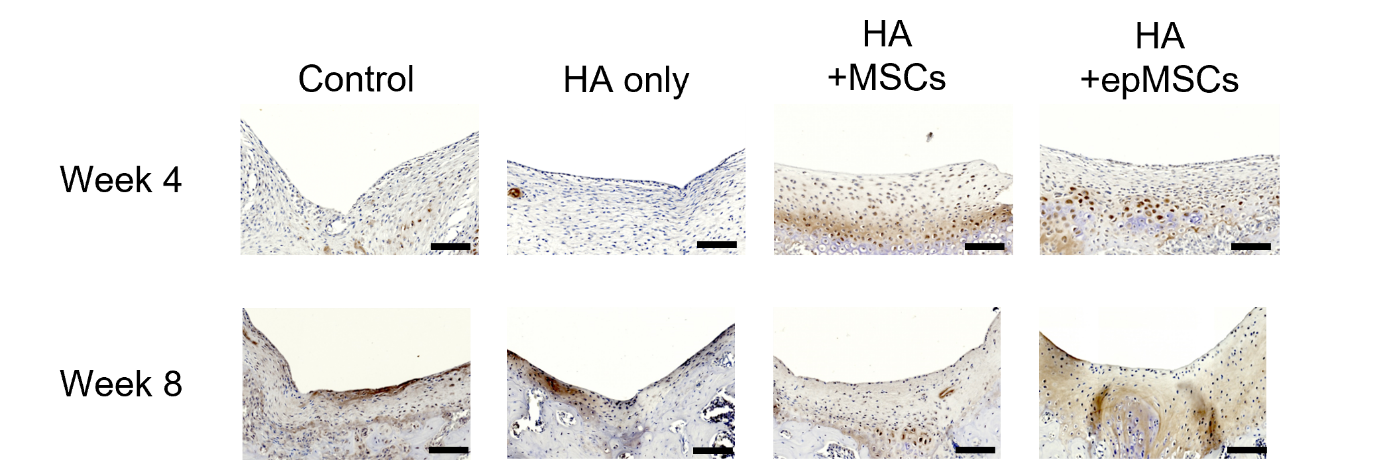


**Fig. S6.** Higher-magnification of COL2 immunohistochemical staining of osteochondral defects at 4 and 8 weeks after surgery and transplantation. Scale bar = 100 μm.


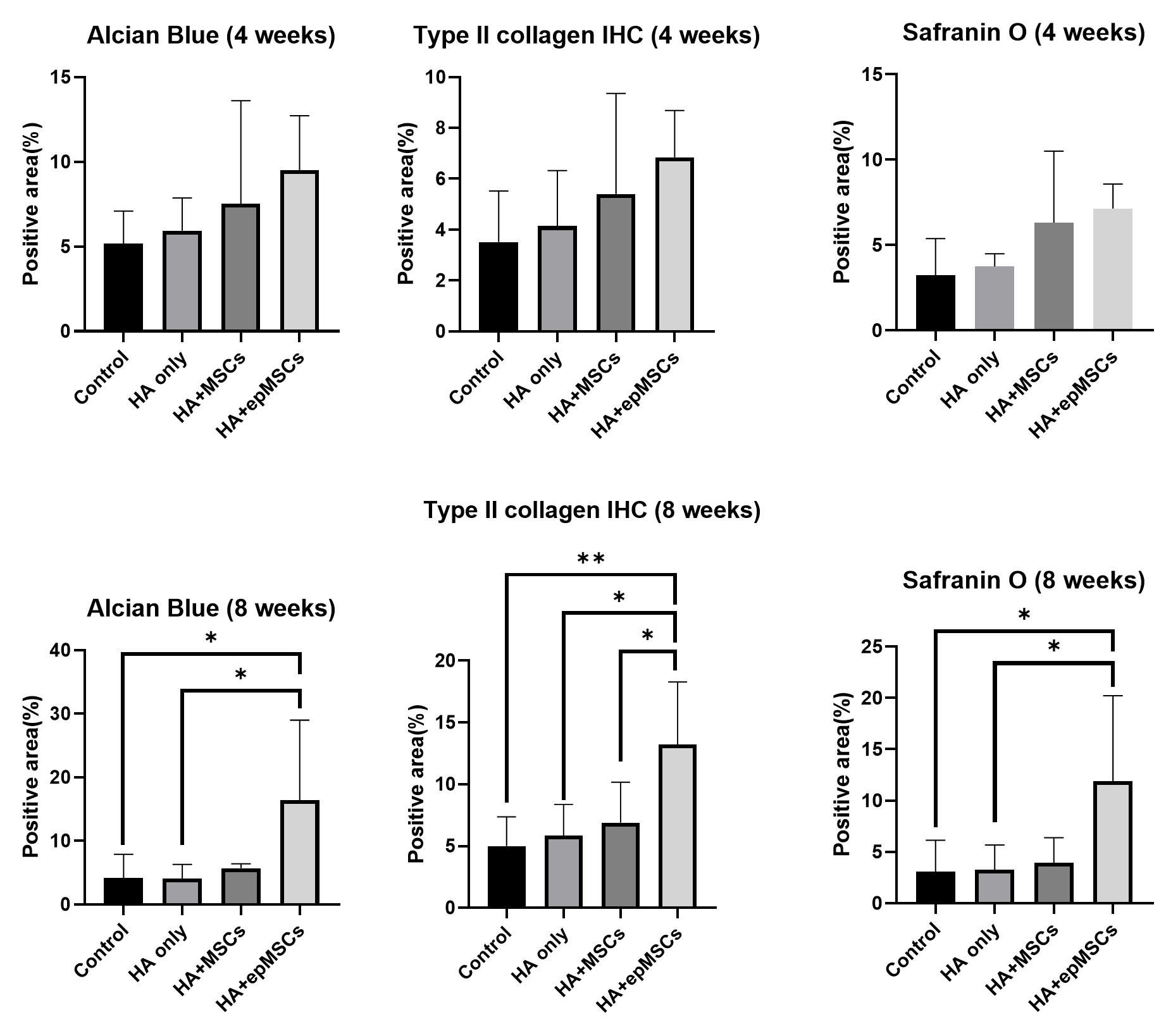


**Fig. S7.** Histological and immunohistochemical analyses of cartilage regeneration at 4 and 8 weeks after surgery. The positive areas of Alcain blue, COL2 IHC, and Safranin O staining were quantified by calculating the ratio of the positively stained area within the regenerated area to the total defect area in the OCD and are represented by the bar graphs (*n* = 5, **p* < 0.05; ***p* < 0.01).

**Table S1.** The International Cartilage Repair Society (ICRS) scoring system for macroscopic assessment for cartilage repair.

| Cartilage repair assessment ICRS | Points |
| --- | --- |
| Degree of defect repair |  |
| In level with surrounding cartilage | 4 |
| 75 % repair of defect depth | 3 |
| 50 % repair of defect depth | 2 |
| 25 % repair of defect depth | 1 |
| 0 % repair of defect depth | 0 |
|  |  |
| Integration to border zone |  |
| Complete integration with surrounding cartilage | 4 |
| Demarcating border < 1 mm | 3 |
| 3/4^th^ of graft integrated, 1/4^th^ with a notable border > 1 mm width | 2 |
| 1/2 of graft integrated with surrounding cartilage, 1/2 with a notable border > 1 mm | 1 |
| From no contact to 1/4^th^ of graft integrated with surrounding cartilage | 0 |
|  |  |
| Macroscopic appearance |  |
| Intact smooth surface | 4 |
| Fibrillated surface | 3 |
| Small, scattered fissures or cracks | 2 |
| Several, small or few but large fissures | 1 |
| Total degeneration of grafted area | 0 |
|  |  |
| Overall repair assessment |  |
| Grade I: normal | 12 |
| Grade II: nearly normal | 11-8 |
| Grade III: abnormal | 7-4 |
| Grade IV: severely abnormal | 3-1 |

**Table S2.** The modified O’Driscoll score for histological assessment for cartilage repair.

| Characteristic | Score |
| --- | --- |
| 1. Hyaline cartilage (%) |  |
| 80 – 100 | 8 |
| 60 – 80 | 6 |
| 40 – 60 | 4 |
| 20 – 40 | 2 |
| 0 – 20 | 0 |
| 1. Structural integrity |  |
| 1. Surface irregularity |  |
| Smooth and intact | 2 |
| Fissures | 1 |
| Severe disruption, fibrillation | 0 |
| 1. Structural integrity |  |
| Normal | 2 |
| Slight disruption, including cysts | 1 |
| Severe lack of integration | 0 |
| 1. Thickness |  |
| 100 % of normal adjacent cartilage | 2 |
| 50 % to 100 % or thicker than normal | 1 |
| 0 – 50 % | 0 |
| 1. Bonding to adjacent cartilage |  |
| Bonded at both ends of graft | 2 |
| Bonded at one end / partially both ends | 1 |
| Not bonded | 0 |
| 1. Freedom from cellular changes of degeneration |  |
| Normal cellularity, no clusters | 2 |
| Slight hypocellularity, < 25 % chondrocyte clusters | 1 |
| Moderate hypocellularity, > 25 % clusters | 0 |
| 1. Freedom from degenerate changes in adjacent cartilage |  |
| Normal cellularity, no clusters, normal staining | 3 |
| Normal cellularity, mild clusters, normal staining | 2 |
| Mild or mod hypocellularity, slight staining | 1 |
| Severe hypocellularity, slight staining | 0 |
| 1. Reconstitution of subchondral bone |  |
| Complete reconstitution | 2 |
| Greater than 50 % reconstitution | 1 |
| 50 % or less reconstitution | 0 |
| 1. Bonding of repair cartilage to de novo subchondral bone |  |
| Complete and uninterrupted | 2 |
| < 100 % but > 50 % reconstitution | 1 |
| < 50 % complete | 0 |
| 1. Safranin O staining |  |
| > 80 % homogeneous positive stain | 2 |
| 40 – 80 % homogeneous positive stain | 1 |
| < 40 % homogeneous positive stain | 0 |
| Total score | Max 27 |
